# Impact of truncating diffusion MRI scans on diffusional kurtosis imaging

**DOI:** 10.1007/s10334-024-01153-y

**Published:** 2024-02-23

**Authors:** Ana R. Fouto, Rafael N. Henriques, Marc Golub, Andreia C. Freitas, Amparo Ruiz-Tagle, Inês Esteves, Raquel Gil-Gouveia, Nuno A. Silva, Pedro Vilela, Patrícia Figueiredo, Rita G. Nunes

**Affiliations:** 1 Institute for Systems and Robotics-Lisboa and Department of Bioengineering, Instituto Superior Técnico, Universidade de Lisboa, Lisbon, Portugal; 2 Champalimaud Research, Champalimaud Foundation, Lisbon, Portugal; 3 Neurology Department, Hospital da Luz, Lisbon, Portugal; 4 Center for Interdisciplinary Research in Health, Universidade Católica Portuguesa, Lisbon, Portugal; 5 Learning Health, Hospital da Luz, Lisbon, Portugal; 6 Imaging Department, Hospital da Luz, Lisbon, Portugal

**Keywords:** Diffusion MRI (dMRI), Diffusion tensor imaging (DTI), Diffusional kurtosis imaging (DKI), Subsampling, Histogram-metrics

## Abstract

**Objective:**

Diffusional kurtosis imaging (DKI) extends diffusion tensor imaging (DTI), characterizing non-Gaussian diffusion effects but requires longer acquisition times. To ensure the robustness of DKI parameters, data acquisition ordering should be optimized allowing for scan interruptions or shortening. Three methodologies were used to examine how reduced diffusion MRI scans impact DKI histogram-metrics: 1) the electrostatic repulsion model (Opt_EEM_); 2) spherical codes (Opt_SC_); 3) random (Random_TRUNC_).

**Materials and methods:**

Pre-acquired diffusion multi-shell data from 14 female healthy volunteers (29±5 years) were used to generate reordered data. For each strategy, subsets containing different amounts of the full dataset were generated. The subsampling effects were assessed on histogram-based DKI metrics from tract-based spatial statistics (TBSS) skeletonized maps. To evaluate each subsampling method on simulated data at different SNRs and the influence of subsampling on in vivo data, we used a 3-way and 2-way repeated measures ANOVA, respectively.

**Results:**

Simulations showed that subsampling had different effects depending on DKI parameter, with fractional anisotropy the most stable (up to 5% error) and radial kurtosis the least stable (up to 26% error). Random_TRUNC_ performed the worst while the others showed comparable results. Furthermore, the impact of subsampling varied across distinct histogram characteristics, the peak value the least affected (Opt_EEM_: up to 5% error; Opt_SC_: up to 7% error) and peak height (Opt_EEM_: up to 8% error; Opt_SC_: up to 11% error) the most affected.

**Conclusion:**

The impact of truncation depends on specific histogram-based DKI metrics. The use of a strategy for optimizing the acquisition order is advisable to improve DKI robustness to exam interruptions.

**Supplementary Information:**

The online version contains supplementary material available at 10.1007/s10334-024-01153-y.

## Introduction

Diffusion magnetic resonance imaging (dMRI) is an imaging modality that provides sensitive biomarkers of brain microstructural properties. Due to its clinically feasible acquisition times, diffusion tensor imaging (DTI) is still the most used dMRI technique [1, 2]. The diffusion tensor can be estimated from data comprising at least a single non-zero b-value and a minimum of six gradient directions (single-shell) together with at least one acquisition with no diffusion-weighting [3, 4]. However, as DTI fails to characterize non-Gaussian diffusion effects, an extension of DTI was proposed—the diffusional kurtosis imaging (DKI) [5]. In addition to all standard DTI parameters, DKI provides diffusional kurtosis parameters that quantify the degree of non-Gaussian diffusion—which were shown to provide unique information of tissue microstructural properties in both healthy and pathologic conditions [5–10]. To properly estimate the full DTI and DKI tensors, several b-values (multi-shell) and a higher number of gradient directions must be sampled: a minimum of 2 non-zero b-values (or shells) and at least 15 non-collinear directions per shell, together with one acquisition with no diffusion-weighting. This comes at the cost of inherently increased acquisition times [11, 12].

In the context of clinical practice and research scenarios, finding a balance between the design of acquisition schemes and patient comfort is often challenging, particularly when studying patients suffering from pathologies that impact their tolerance to MRI scanning (e.g., stroke, cancer, neurodegenerative diseases, migraine), and even more when performing longitudinal (multi-session) studies with strict time limitations. To provide an example, let us consider the possible constraints of a two-sessions study with migraine patients in which we aim to acquire a multi-shell dMRI sequence within an hour-long multimodal MRI protocol, when patients are experiencing symptoms (headache, nausea, photophobia, etc)—session A and without symptoms—session B. The current scenario requires a fundamental trade-off between protocol duration, data collection and patients’ tolerance, especially during session A. If patients’ tolerance permits and there are no time restrictions, it would be preferable to use a complete protocol. However, in cases when an interruption in the acquisitions is necessary, a shortened protocol should be carefully considered to optimize the collection of information and ensure cross-exam comparability. Hence, it is important to ensure that an abbreviated protocol, if applied in one of the sessions, remains compatible with full protocols in subsequent or previous sessions for the same subject. Therefore, it is particularly important to design acquisition schemes that still produce precise and accurate DKI parameter estimates if the acquisitions need to be interrupted [13–19]. It was suggested for clinical use that the optimal sampling scheme for kurtosis estimates should include three shells: 0, 1000, and 2000 s/mm^2^; and at least 20 gradient directions per shell [20, 21]. It was also reported that DKI parameters are less dependent on the number of acquired b-values when the maximum b-value is held constant [22, 23]; however, they can be more influenced by the number of sampled diffusion orientations [17, 21].

After data collection, several additional processing steps may also have an impact on the accuracy of estimated parameters when using incomplete scans. To assess white matter (WM) microstructural alterations, researchers employ region-of-interest (ROI) analysis for local assessment or compute histogram-based metrics across the brain for a more comprehensive evaluation. Both approaches have demonstrated their value in detecting differences between groups and relationships with clinical variables. Nevertheless, these analyses may considerably be influenced by different diffusion MRI acquisition parameters, such as the exact diffusion gradient directions and b-values used [14]. This concern becomes particularly pertinent when dealing with diffusion MRI datasets from incomplete scans, as the acquired acquisition parameters may not uniformly cover their parameter domain. However, this issue can be mitigated by optimizing the acquisition parameter sampling and ordering [13, 16–18, 24, 25].

The state-of-the-art techniques for generating gradient directions for multi-shell acquisitions typically follow the generalization of the bipolar electrostatic repulsion model (considering all individual gradient directions and their negative)—electrostatic repulsion model (EEM) [19, 26]. The ordering by which the selected gradient directions are acquired can then be optimized using brute-force search to find a global optimum that reduces the impact of data truncation in case data acquisition is interrupted [13, 15, 27]. While this model was introduced with the implicit aim of maximizing the angular distance between sampled directions, the typical electrostatic energy cost function is influenced by angular distance but does not explicitly measure it [28]. Other approaches were proposed to generate or subsample sampling schemes based on the maximization of the angular resolution between samples (i.e., spherical codes) [29]. While both approaches for generating sampling schemes were proven to perform similarly in terms e.g., of angular error of the reconstructed fibre directions in WM [19, 29], their impact on DKI parameters and associated distribution characteristics (e.g., median, histogram peak height, width, and values) have not been fully explored.

In this study, we aim to evaluate a strategy for choosing and ordering a subset of gradients in DKI acquisitions, from a complete protocol that was previously optimized to balance scan duration, parameter estimation accuracy, and cross-session comparability. The impact of truncating dMRI data from different ordering strategies are investigated on histogram-based DKI metrics.

## Methods

All participants gave written informed consent in accordance with the Declaration of Helsinki after the study was approved by the local Ethics Committee.

### dMRI sampling scheme and subsampling strategies

**Fig. 1:**
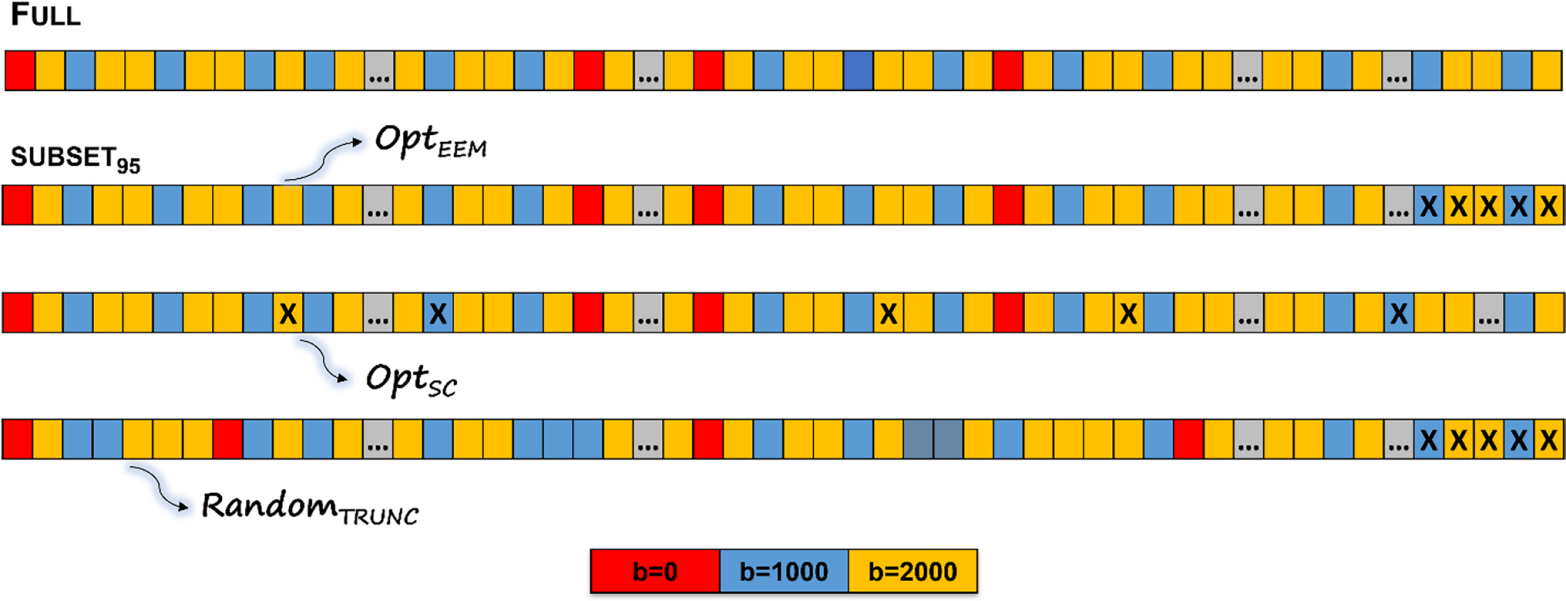
The subsampling process is shown schematically. Based on the electrostatic repulsion model [28], the optimal sampling scheme was first designed to comprise three shells (*b* = 400 s/mm^2^, *b* = 1000 s/mm^2^ (blue), *b* = 2000 s/mm^2^ (yellow)) spread equally around the sphere along 32, 32, and 60 gradient directions; and 8b0s (red). Although the original sampling scheme included 3 b-values, we focused on *b* = 1000, 2000 s/mm^2^ shells to evaluate DKI parameters. This diagram depicts the procedure for discarding 5% (subset95) of the total number of volumes per shell (represented by X) using three different strategies, applied to the current full-length protocol (1) Opt_EEM_ (subsamples the original scheme by removing volumes from the end of it); (2) Opt_SC_ (subsamples the original scheme by maximizing the angular resolution, which results in removing gradient directions across the sampling scheme – in a real scenario, these volumes would have been at the end of the full protocol); and (3) Random_TRUNC_ (randomly shuffles the initially optimized scheme and applies truncation)

We started by generating an optimized multi-shell sampling scheme using the *gen_scheme* script from MRTrix (mrtrix.org) considering a protocol comprising: 3 diffusion-shells (uniformly distributed over the sphere) with *b* = 400, 1000, 2000 s/mm^2^ along 32, 32, 60 gradient directions, respectively; and 8 non-diffusion-weighted volumes. Although the original sampling scheme included a 3 b-values (3 shells), our analysis is focused on the gradient directions for *b* = 1000, 2000 s/mm^2^ and eight *b* = 0 s/mm^2^ repetitions to assess the impact of scan interruptions in the higher b-values typically acquire for DKI reconstruction. All the experiments for these selected b-values are used as the reference complete (full) scheme for both simulations and in-vivo data collection. We then tested three different approaches for shortening the protocol (Fig. 1). The first method (Opt_EEM_) would correspond to direct truncation of the original scheme, since the *gen_scheme* script incrementally adds new volumes using a brute-force approach to optimize the order of the gradient directions—the aim is to achieve a uniform distribution over the sphere as evaluated by the bipolar EEM. This is done while keeping the same relative proportion of directions for each shell in case the acquisition is interrupted (equivalent to truncating the original full-length scheme). The two other tested approaches would require that the diffusion scheme would first be reordered prior to being used for scanning, when truncation could occur. The Opt_SC_ method directly maximizes the smallest angle difference between two sample locations on the unit sphere, i.e., the covering radius that measures the angular resolution (contrarily to EEM). In this study, we consider an initial sampling scheme optimized by the conventionally used EEM model followed by the selection of specific gradients based on the maximization of the angular resolution. Finally, for a reference, we also consider an approach in which the original full scheme is randomly shuffled and then truncated the data (Random_TRUNC_). To generate the sampling schemes corresponding to reducing the number of samples per shell using the Opt_SC_ method [26, 29], we use the code available at (https://diffusionmritool.github.io/demos/demo_separate_HCPQ390x3_30x3.html). It should be noted that the Opt_SC_ sampling scheme for each subset is obtained sequentially (for example, the sampling scheme generated by reducing 5% is utilized as an input to get the sampling scheme equivalent to excluding 10% of the number of samples and so on). The idea is that the diffusion volumes that would be removed for each subsampling level would have been acquired last if the previous subsampling level had been applied instead. Finally, we generate six optimal data subsets using both methods by excluding 5, 10, 20, 30, 40 or 50% of the number of volumes per shell (Table 1, Fig. 1).

**Table 1:** Description of the total number of gradient directions acquired per shell (*b*=1000, and *b*=2000 s/mm^2^), for the complete (full) dataset and derived subsets (each dataset includes 8 non-diffusion volumes)

Dataset	*b* = 1000 s/mm^2^	*b* = 2000 s/mm^2^
Full	32	60
Subset95	30	57
Subset90	29	54
Subset80	26	48
Subset70	22	42
Subset60	19	36
Subset50	16	30

### Simulations

To aid in the interpretation of the in vivo results, we also evaluate the impact of each subsampling method on the estimation of each diffusion parameter derived from diffusion-tensor (i.e., fractional anisotropy (FA), mean diffusivity (MD), axial diffusivity (AD), and radial diffusivity (RD) maps), and kurtosis-tensor, (i.e., mean kurtosis (MK), axial kurtosis (AK), and radial kurtosis (RK) maps), we firstly perform Monte-Carlo simulations of a single-voxel which are representative of WM voxels. Rician noise is introduced to the synthetic diffusion-weighted signals to achieve different signal-to-noise (SNR) levels (SNR=10, 20, 30, 40, 50) in the b0 volumes. To demonstrate ground truth convergence, a higher SNR level (SNR=1000) is employed as a reference. The signals are generated based on the same ground truth values of the diffusion and kurtosis tensors used in previous studies [30, 31] which represent tissue as a mixture of two fibers crossing at 60 degrees. For each fiber, two tensors are considered to represent intra- and extra-cellular environments with equal fractions. For the intra-cellular component, the AD and RD are set to 1.4×10^−3^ mm^2^/s and 0.1×10^−3^ mm^2^/s, respectively. The corresponding diffusivities for the extra-cellular compartment are set to 2.0×10^−3^ mm^2^/s and 0.5×10^−3^ mm^2^/s. This is implemented as exemplified in voxel case 4 at https://github.com/dipy/dipy-dki-paper/blob/master/Figures_simulations_noise_free.ipynb [31]. Diffusion-weighted signals are simulated for the gradient directions of *b* = 1000, 2000 s/mm^2^ and eight *b* = 0 s/mm^2^ images repetitions according to the reference full scheme (i.e., total of 100 volumes). Then the synthetic signals for each derived subset are obtained by using the three subsampling methods to extract the corresponding reduced gradient schemes from the original full-length scheme. These simulations are implemented in Python using tools from diffusion imaging in Python (DIPY) package [30, 32]. The experiments are repeated for 100 different diffusion-tensor orientations and 100 noise instances for each tensor orientation (i.e., total of 10000 simulation instances). The code used to generate the simulations performed in this paper is available at https://github.com/LaSEEB/dMRI-subsampling_sims.git. Then we use DESIGNER [33] to perform constrained tensor fitting by restricting all apparent directional kurtosis to positive values, which is more robust when applied to low SNR images.

Finally, a 3-way repeated-measures ANOVA is performed to compare the effects of each subsampling method at different SNR conditions and on each diffusion parameter. The analyses are carried out using the JASP software (version 0.16.1.0) with the following within-subjects factors: (1) Method (Opt_EEM_; Opt_SC_ and Random_TRUNC_); (2) Subset (full, subset95, subset90, subset80, subset70, subset60, subset50); and (3) SNR varied from 10 to 50. When interactions between Method and Subset and/or SNR are identified as being statistically significant, a post-hoc analysis is performed to identify significant pairwise comparisons by applying paired t-tests. Bonferroni correction is used to account for multiple comparisons and significant effects are considered for adjusted *P*<0.05.

### Human brain data

A group of 14 healthy women (29 ± 5 years) was recruited to undergo MRI scanning using the full multi-shell sampling scheme protocol, as part of an ongoing study focused on episodic migraine, including longitudinal evaluations of both groups. Whole-brain multi-shell dMRI images were obtained using a 3T Siemens Vida scanner with a 64-channel radio-frequency receive head coil using 2D echo-planar imaging (EPI) sequence: TR/TE=6800/89 ms, 66 contiguous slices in-plane GRAPPA factor 2, simultaneous multi-slice (SMS) factor 3, 2 mm isotropic resolution. Additionally, we also acquired 3 volumes with opposite phase-encoding to correct b0-related distortions.

The original fully sampled datasets were pre-processed using the DESIGNER pipeline [33]. Firstly, denoising [34], Gibbs ringing correction [35]; and Rician bias correction [36] were performed using MRTrix tools [27]. Secondly, FSL was employed for B0-related and eddy-current geometric distortions, as well as motion [37–39]; and thirdly bias field correction was performed with *-ants* option using MRTrix. Then, the subsets were generated using Python from the pre-processed diffusion MRI data to improve computational efficiency, given that pre-processing independently after generating the subsets has shown reduced impact (data not shown). Since our simulations had revealed that Random_TRUNC_ performed worse than Opt_EEM_ and Opt_SC_, *in vivo* subsets were only produced for these two techniques. For each dataset and corresponding subsets, DKI fitting was performed using the unconstrained DESIGNER’s DKI fitting to derive maps for 7 parameters: FA, MD, AD, RD, MK, AK and RK, since this fitting strategy was shown to produce more robust estimates for the SNR levels for typical *in vivo* diffusion MRI data [33]. For each level of subsampling and diffusion parameter, skeletonised maps were computed using FSL’s tract-based spatial statistics (TBSS) standard pipeline [40], where the mean WM skeleton was thresholded at 0.3 [41].

Normalized histograms (number of bins: 1000; divided by the total number of voxels and bin width) of each diffusion parameter were computed across skeletonized maps using R (r-project.org/) to evaluate the effects of subsampling [41]. Then, we extracted from each histogram the following characteristics: median, peak height, peak value, and peak width (difference between 95th and 5th percentiles [42]). Finally, to test the effects of Method (Opt_EEM_, Opt_SC_, and Random_TRUNC_) and Subsampling (full, subset95, subset90, subset80, subset70, subset60, subset50) on each diffusion parameter and metric, we used a 2-way repeated measures ANOVA (Bonferroni correction, *P*<0.05), on JASP (jasp-stats.org/).

## Results

### Simulations

**Fig. 2:**
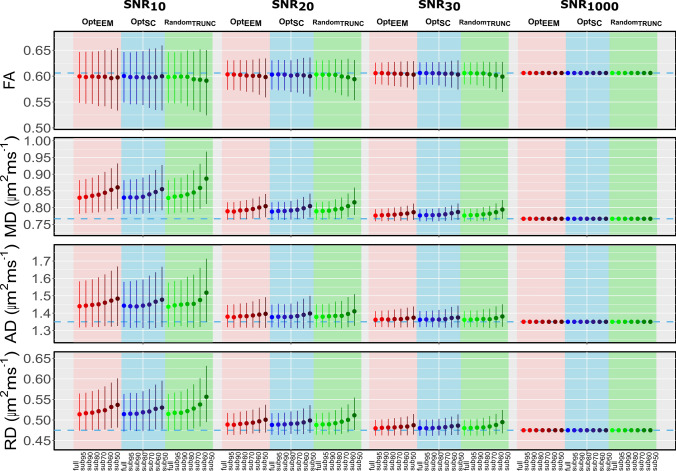
Median and interquartile range of DKI diffusion parameters derived from simulated signals are displayed as a function of SNR. Median and interquartile range (over repetitions) of FA, MD, AD and RD parameters obtained from the full and subsampled data (subset95; subset90; subset80; subset70; subset60; and subset50) using the following methods: Opt_EEM_ (in red), Opt_SC_ (in blue), and Random_TRUNC_ (in green), shown for multiple SNR levels (snr10; snr20; snr30; and snr1000); corresponding ground truth values are shown by a dashed blue line

**Fig. 3:**
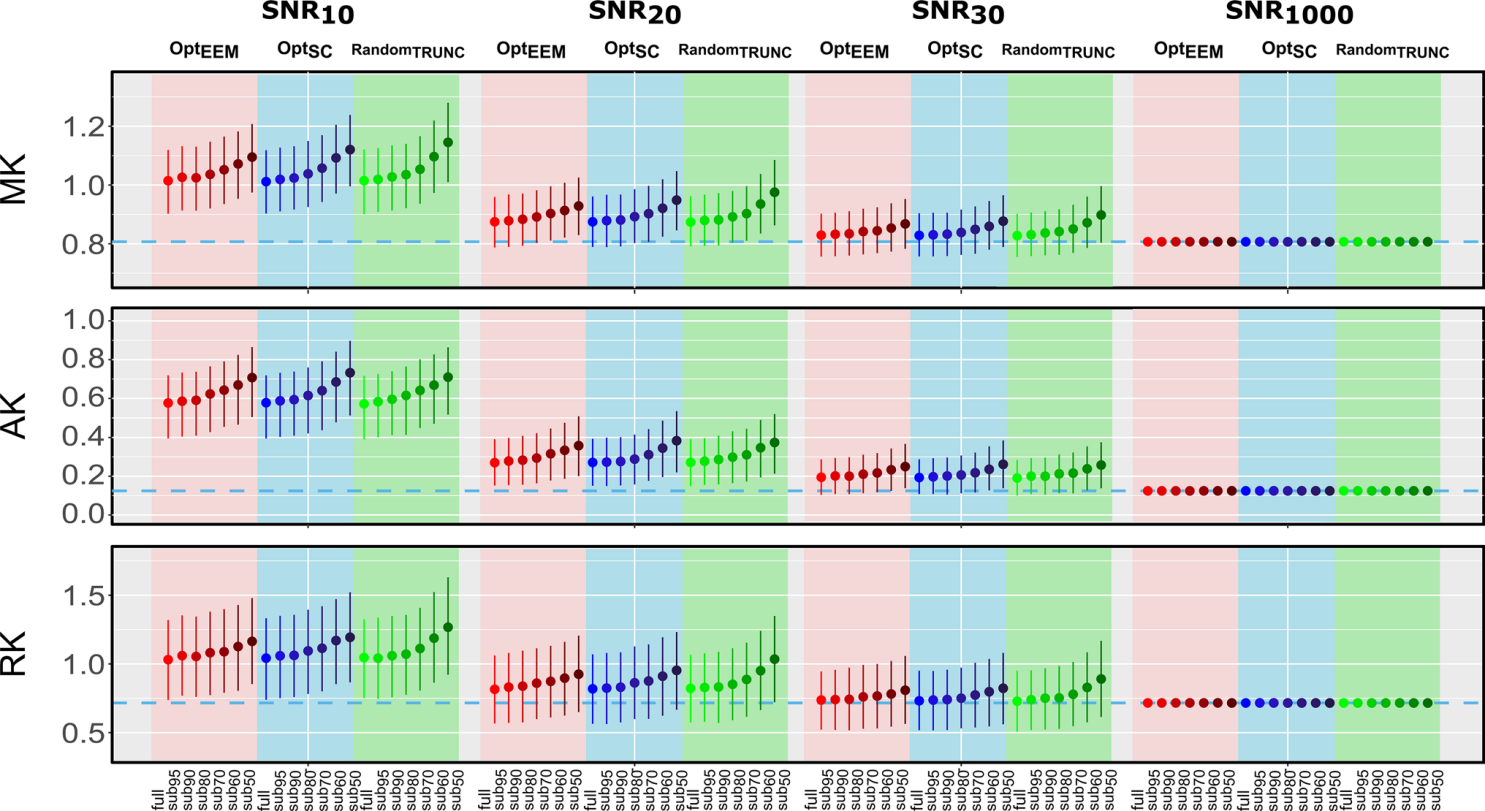
Median and interquartile range of DKI kurtosis parameters derived from simulated signals are displayed as a function of SNR. Median and interquartile range of MK, AK and RK parameters obtained from the full and subsampled data (subset95; subset90; subset80; subset70; subset60; and subset50) using the following methods: Opt_EEM_ (in red), Opt_SC_ (in blue), and Random_TRUNC_ (in green) are shown for multiple SNR levels (snr10; snr20; snr30; and snr1000); corresponding ground truth values are shown by a dashed blue line

Figures 2 and 3 show the median and interquartile ranges of the different diffusion (i.e., FA, MD, RD and AD) and kurtosis (i.e., MK, AK and RK) parameters, respectively, derived using the three subsampling methods: Opt_EEM_; Opt_SC_ and Random_TRUNC_, as a function of SNR. The simulations demonstrate that subsampling has a significant impact on all diffusion and kurtosis parameters depending on the subsampling method, percentage of discarded volumes and level of SNR. Particularly, Figs. 2 and 3 show that the median values of estimated diffusion and kurtosis parameters deviate from their corresponding ground truth values as SNR decreases, likely due to Rician noise biases. Analogous behavior is observed for SNR 40 and 50, but not shown for simplicity. To inspect the effects of subsampling independently to Rician biases, the relative error computed as the differences between the metric derived from each subset and the full dataset are also shown in Supplementary Figs. S1 and S2.

Table 2 displays an overview of the statistically significant results from the post-hoc analyses, in comparison with the fully sampled metrics to assess the influence of the method and subsampling percentage. Table S1 shows the corresponding relative difference error (in percentage) for all diffusion parameters and subsampling methods at a common SNR of 20 (Supplementary Material). For all diffusion parameters, three-way ANOVA results reveal significant main effects of: Method, Subsampling, and SNR (*P* < 0.001). Furthermore, significant interactions between all factors are also found (*P* < 0.001). We observe that as the level of subsampling increased and the SNR level decreased, more biased estimates are produced for all methods. However, the different diffusion and kurtosis parameters are affected differently. Overall, the subsampling level has the least influence on FA estimates across all SNR levels and methods.

**Table 2:**
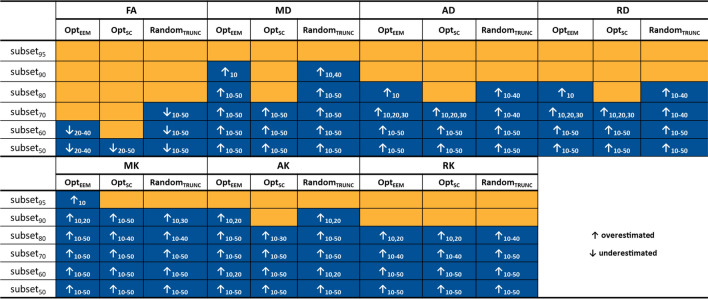
Summary of the pairwise comparisons between metrics extracted from subsampled and fully sampled data. Statistically significant differences are highlighted by the arrows in the blue cells. For example, this table shows that FA estimates decreased compared to the fully sampled protocol when the data was subsampled with Opt_SC_ and only 50% of the data (subset50) was used at noise levels ranging from 20 to 50. Although FA values were similarly underestimated (↓) by Opt_EEM_, there was a significant difference between subset50 and subset60 and the fully sampled protocol, at noise levels ranging from 20 to 40. Finally, at SNR values ranging from 10 to 50, the non-optimized sampling method (Random_TRUNC_) revealed greater sensitivity to subsampling (subset50, subset60, and subset70). Method = Opt_EEM_, Opt_SC_, Random_TRUNC_; Subsampling = subset95, subset90, subset80, subset70, subset60, subset50; SNR = 10, 20, 30, 40, 50, 1000; ↑ Overestimated; ↓ Underestimated.

From these results, we can observe that FA values are underestimated in specific conditions. For instance, when using Opt_SC_ and keeping only 50% of the data, FA is underestimated at SNR levels ranging from 20 to 30 (Figs. 2, 3, and Table 2). FA is also underestimated within the same SNR range, when using Opt_EEM_ and subsampling over 40%. On the other hand, when Random_TRUNC_ method (non-optimized) is considered, we found that the FA is already biased for subsampling exceeded 30%, for all the SNRs considered.

Regarding MD, we observe consistent increases as the number of discarded volumes increased for all methods (Figs. 2 and 3). Specifically, our statistical analysis in Table 2 reveals biased estimates for subsampling levels over 10% (Opt_EEM_; SNR: 10–50), 30% (Opt_SC_; SNR: 10–50), and 10% (Random_TRUNC_; SNR: 10–50). The bias is greatly increased when Random_TRUNC_ is used, 4%, relative to when the Opt_SC_ and Opt_EEM_ estimation methods are employed: 1.9% and 2%, respectively (SNR=20; subsampling of 50%). Moreover, AD and RD are overestimated compared to the full-length scheme for subsampling over 30% (Opt_SC_; SNR: 10–50); and over 20% for both Opt_EEM_ and Random_TRUNC_ (SNR: 10–50).

Compared to diffusion parameters, kurtosis parameters exhibit a greater dependence on the subsampling level and SNR (Figs. 2 and 3). Subsampling just 10% of data (subset90) is shown to be enough to produce overestimated MK estimates when using Opt_SC_ and Random_TRUNC_, whereas removing merely 5% of the data (subset95) already affects the estimates when using Opt_EEM_ (Table 2). A large bias is observed with respect to the fully sampled data when MK values are estimated from 50% of the data (subset50) using Random_TRUNC_ 11% compared to 8% when using Opt_SC_ and 7% when using Opt_EEM_ (SNR=20).

### Human brain data

**Fig. 4:**
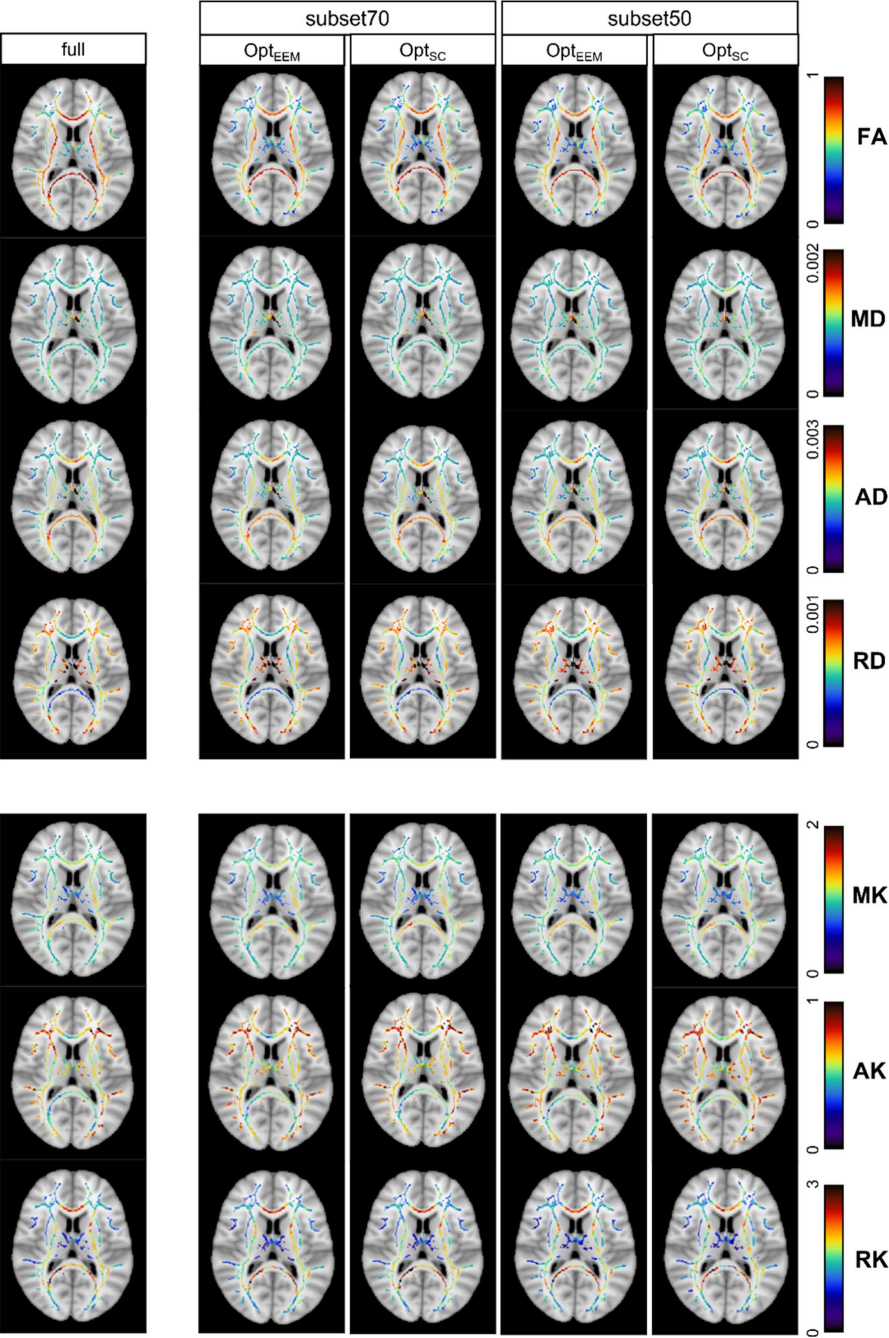
Examples of axial slices showing skeletonised DTI (FA, MD, AD and RD) and DKI (MK, AK and RK) maps obtained from the fully sampled data (on the left) and two different subsampling approaches (Opt_EEM_ and Opt_SC_) for two representative subsampling levels (subset70 and subset50). MD, AD and RD maps are in mm^2^s^−1^

**Fig. 5:**
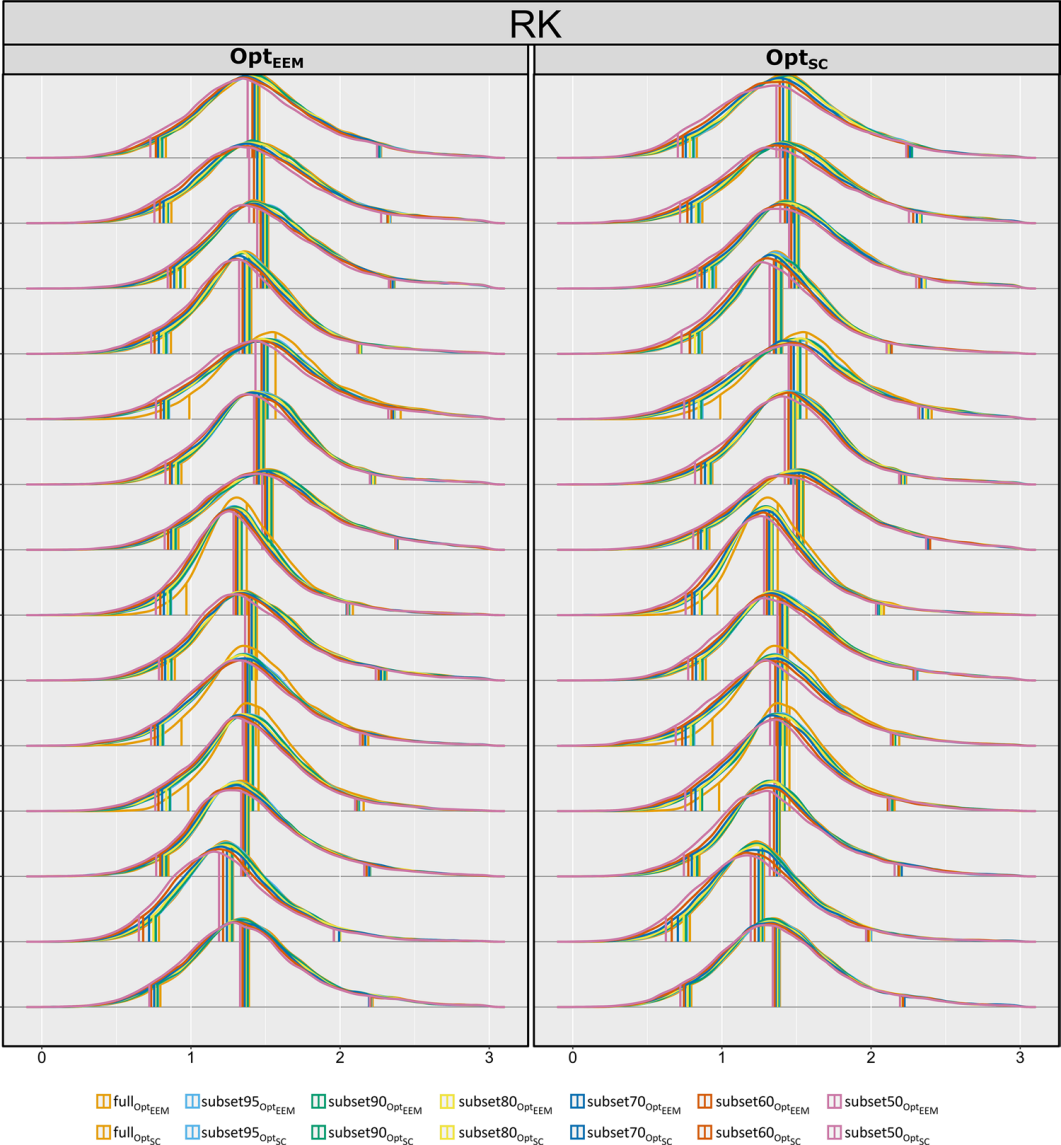
Illustrative examples of overlapping whole-brain histograms derived from fully and subsampled RK maps using both methods: Opt_EEM_ (in red) and Opt_SC_ (in blue); black lines highlight the histogram-based metrics (median and peak width). Each row corresponds to a different subject

**Fig. 6:**
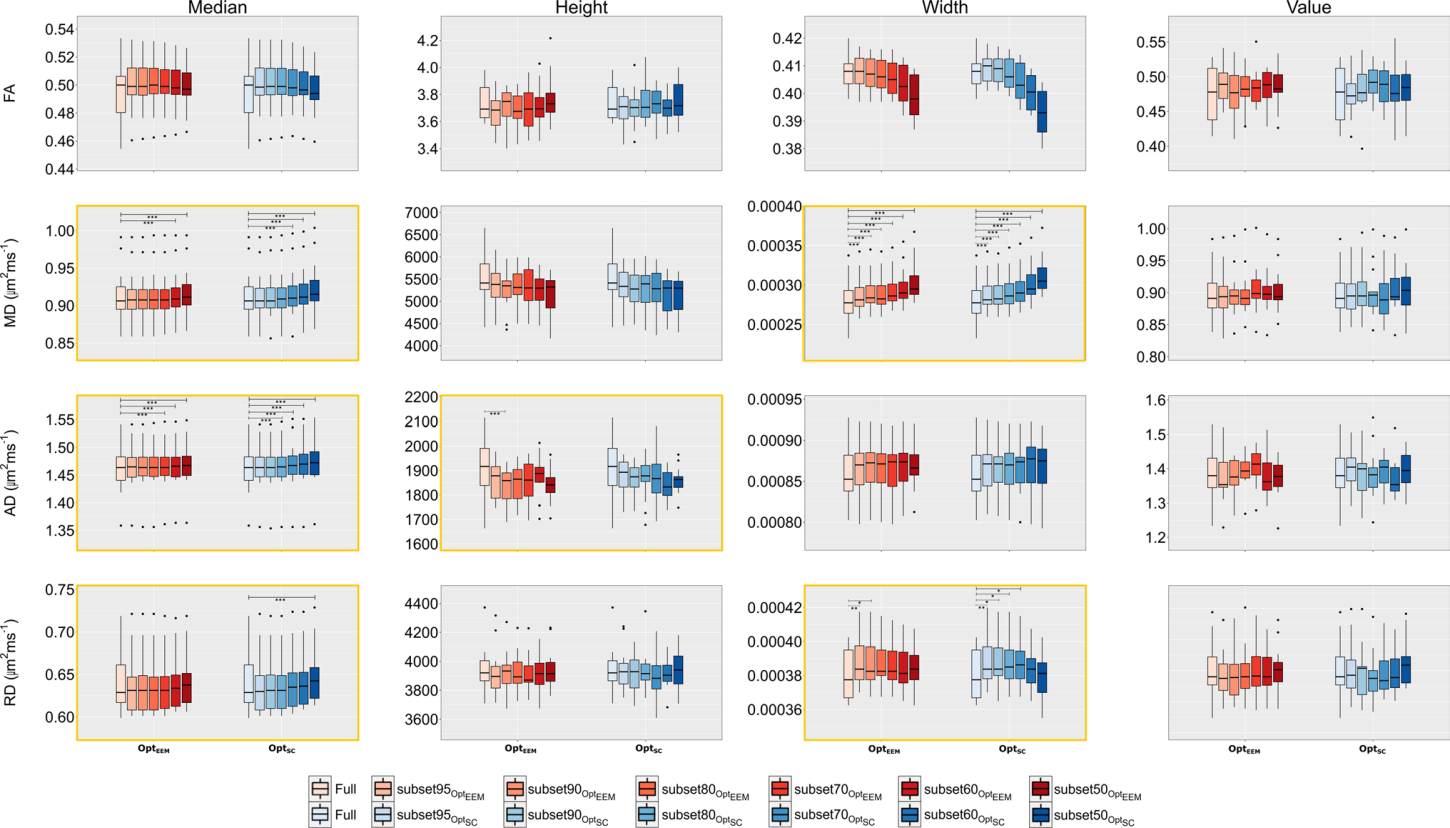
Boxplots showing the distributions of FA, MD, AD and RD maps histogram-metrics: median, peak height, peak width and peak value; which were obtained from the full and corresponding subsampled data (subset95; subset90; subset80; subset70; subset60; and subset50) using both methods: Opt_EEM_ (in red) vs Opt_SC_ (in blue). Metrics showing significant interactions are highlighted by yellow boxes

**Fig. 7:**
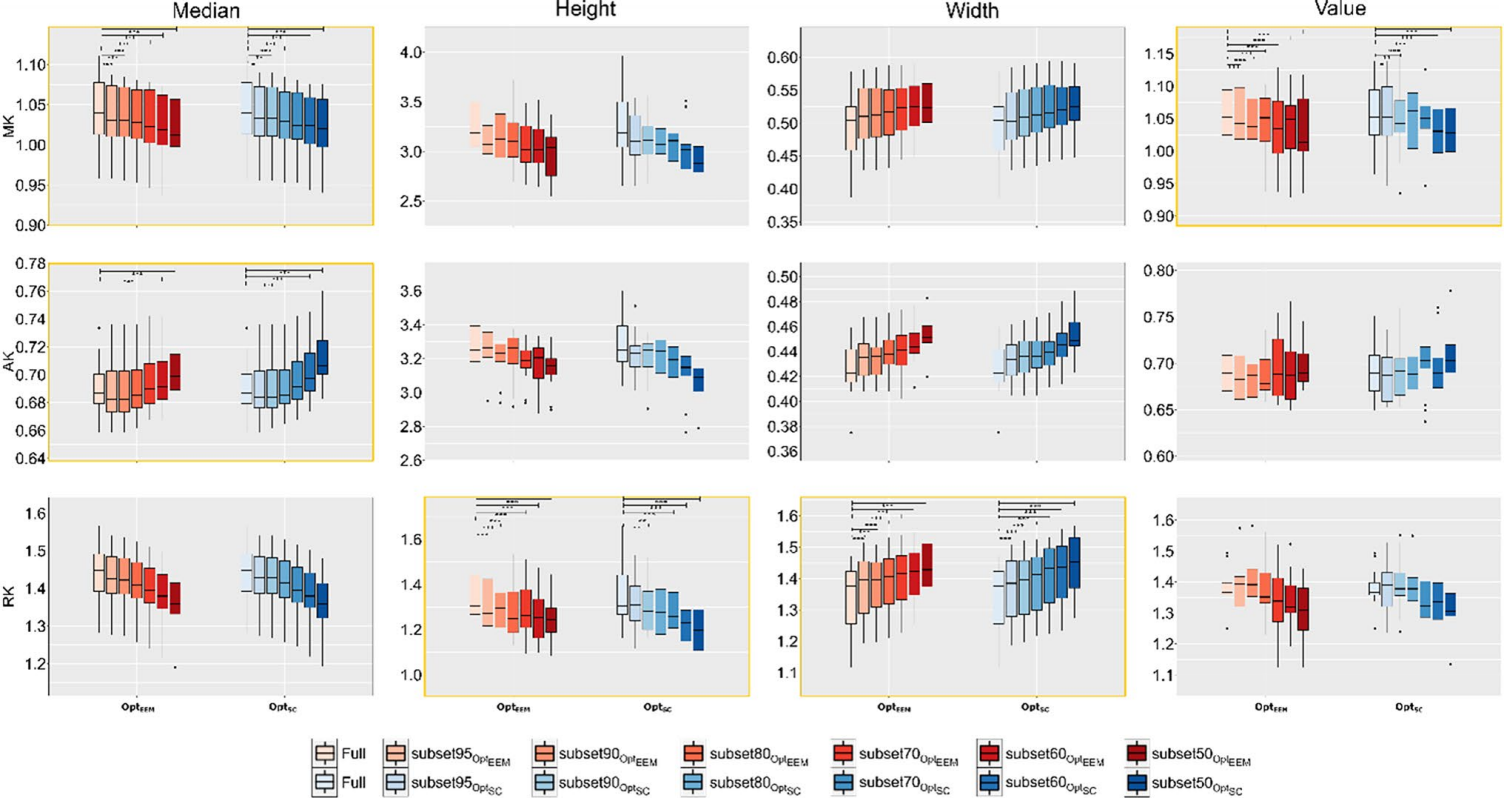
Boxplots showing the distributions of MK, AK and RK maps histogram-metrics: median, peak height, peak width and peak value; which were obtained from the full and corresponding subsampled data (subset95; subset90; subset80; subset70; subset60; and subset50) using both methods: Opt_EEM_ (in red) vs Opt_SC_ (in blue). Metrics showing significant interactions are highlighted by yellow boxes

Figure 4 shows the skeletonised DKI parameter maps derived from full, subset70 and subset50 datasets of a representative subject, while Fig. 5 shows overlaid histograms for the most impacted DKI parameter by data subsampled (i.e., RK) for the full dataset and data subsets—left, right for Opt_EEM_ and Opt_SC_ subsampling methods, respectively. The overlaid histograms of FA, MD, AD, RD, MK, and AK are shown in Supplementary Figures S3-S8. For the full dataset and its subsets, boxplots of histogram characteristics for the diffusion and kurtosis parameters computed across subjects are shown in Figs. 6 and 7, respectively. These figures show that data subsampling can affect most of the histogram-based DKI metrics characteristics. Indeed, our results indicate that significant main effect of subsampling effects all histogram-based DKI metrics are present with exception for FA peak height, FA peak value; AD peak value; and RD peak height (as also supported by supplementary information Table S2). In fact, for such metrics, we found no significant main effect of Method, indicating that these metrics are robust to both subsampling level and method (Supplementary Table S2). On the other hand, all histogram metrics for kurtosis parameters show a main effect of subsampling, being, therefore, more susceptible to subsampling bias than the histogram metrics for diffusion parameters.

Regarding the different sampling methods, several histogram-based DKI metrics showed a significant main effect of Method, namely: FA median and peak width; MD median, peak height and peak width; AD and RD median; MK median and peak value; AK median and peak height; and RK peak height (see Supplementary Table S2). A larger number of significant effects of the subsampling method could be detected for the diffusion parameters compared to the kurtosis parameters. Moreover, we also found significant interactions for the following histogram-based DKI metrics: FA and MD median and peak width; AD median and peak height; RD median, peak width and peak value; MK median and peak value; AK median; and RK peak height and peak width. This implies that the effect of subsampling depends on the method applied, particularly for certain histogram-based DKI metrics. In contrast to our simulations, post hoc pairwise comparisons reveal that Opt_SC_ provided more biased estimates for most measures than Opt_EEM_. Remarkably, we found that all histogram characteristics for FA are fairly stable regardless of the method and subsampling percentage. Indeed, subsampling had no effect on FA peak height or peak value, regardless of the method. Despite not being statistically significant after multiple comparison correction, we observe a tendency in FA median and peak width for decrease when comparing the fully sampled and subsampled data. Nonetheless, for all histogram characteristics for FA, their values deviate from the values of the fully sampled data by up to a maximum 5% error (see Table S3 in Supplementary Material). The magnitude of deviations from the fully sampled data reference are summarized for all histogram characteristic, diffusion parameters and sampling methods in Tables S3-S9 (Supplementary Material).

## Discussion

DKI has shown to be more sensitive than the conventional DTI to brain microstructural alterations in several conditions (e.g., brain development and aging [8, 43], mild traumatic injury [44–46] or ischemic stroke [47, 48]. DKI typically requires, however, longer acquisition times than DTI, making this technique more prone to possible scan interruptions. While techniques are used to minimize the impact of using incomplete acquired data in diffusion MRI by optimizing the acquisition parameter sampling and ordering [13], their specific implications on the properties of histogram distributions from DKI parameters have been poorly explored. In this study, the impact of data subsampling on the estimates of histogram metrics extracted from DKI parameters using different methods is evaluated in both simulations and empirical human brain datasets.

Numerical simulations are first used to evaluate the sensitivity of different DKI parameters to subsampling percentage and methods at different SNR levels. Our simulations suggest that the absolute biases in DKI diffusion and kurtosis parameters increase with the level of subsampling and with decreasing SNR. As the SNR increases, the values obtained for all diffusion parameters approach the ground-truth value and the impact of subsampling is reduced. For a typical SNR of 20, kurtosis parameters reveal lower reliability with increasing levels of subsampling, presenting larger percentage biases compared to diffusion parameters. This effect is expected given that the estimation of kurtosis parameters is known to be more sensitive to noise than for the estimation of diffusion parameters [14, 23, 49], which could cost in spatial resolution for DKI [50]. However, these simulations showed that by shortening the acquisition by 30 % (subset70) most of the errors are under 10% for all DKI parameters and methods.

### Main findings

Our simulations also confirmed that subsampling diffusion MRI data by using Opt_EEM_ and Opt_SC_ is preferable to randomly selecting gradient directions over the sphere. This result is in line with previous studies showing that methods for optimal acquisition ordering led to improved DTI and q-ball imaging reconstructions in partially acquired diffusion MRI data [13, 19, 24, 25]. Here, this effect is shown for the first time for DKI parameter estimates. Our simulations also reveal that Opt_SC_ may produce less biased DKI estimates than Opt_EEM_.

Then, we evaluated the subsampling effects on *in vivo* data by extracting histogram-based DKI metrics from skeletonized diffusion and kurtosis parameters: median, peak height, peak width, and peak value. Our results reveal that subsampling effects may have different effects on distinct histogram characteristics. For instance, while the median, peak width, and value of FA histograms are shown to be relatively stable across subsampling levels, its peak width show a much larger dependence with the subsampling levels.

Regarding the subsampling effect across different DKI parameters, our *in vivo* results are in line with the results from simulations. For example, the profiles of FA median and peak width changes as function of the subsampling level presents a pattern analogous to the FA median and interquartile range differences across different subsampling levels observed in the simulation (i.e., decrease with subsampling level). Results for the diffusion parameters (FA, MD, AD, and RD) extracted from DKI are also consistent with previous research. As in Hutchinson et al. [14], we observed a minimal dependency of FA with data subsampling [14]. For a typical SNR value of 20, the FA extracted from the simulated signal exhibited a significant bias, with a maximum inaccuracy of 3% for subsets keeping 70, 60, and 50% of the data. This supports previous reports showing that at least 20 distinct sampling directions (see Table 1) are required for a robust diffusion anisotropic estimate of anisotropy [15]. For the other diffusion parameters (MD, AD, and RD), biases observed for lower subsampling degrees (c.f. Table 2) are consistent with studies demonstrating that a larger number of directions is required to produce accurate estimates when these are not uniformly sampling the unit sphere [15].

Regarding the kurtosis parameters (MK, AK, and RK), results from *in vivo* data confirms that these parameters are more prone to the degree of data subsampling than their diffusion counterparts (MD, AD, RD), with RK being the least stable parameter. Despite this, our *in vivo* results shows that most of histogram characteristic for DKI metrics are relatively stable for up to 10–20% of data subsampling when Opt_EEM_ and Opt_SC_ subsampling methods are used. These results encourage, therefore, the use of acquisitions schemes with optimal acquisition ordering for DKI reconstruction as well as the use of histogram-based analysis in studies in which data acquisitions are prone to scan interruptions. While we do not expect reordering approaches to be able to mitigate the impact of motion in patient populations more likely to exhibit occasional random motion leading to rejection of individual volumes, our results suggest that kurtosis parameters would be more affected by data subsampling resulting from outlier rejection. Given this, the biases reported here probably represent the lower bounds for motion impact corresponding to the best-case scenarios of the least––damaging data rejection, further investigations would be required to confirm this hypothesis.

### Limitations and future studies

When comparing simulations and *in vivo* results, it is important to bear in mind that the variance observed has different sources—while the variance in simulations only reflects the impact of noise, variance in the boxplots for the human data also captures biologic differences across subjects. Furthermore, the histograms were extracted from the TBSS skeleton built for this specific sample. As the skeleton aims to characterize the center of the major white matter pathways, and its construction involves a search along a wider region, this may potentially explain why the *in vivo* FA median values were very stable across all considered subsampling levels.

In addition to the technical difficulties that can arise when scanning patients, it is also important to devise strategies to prevent the loss of the entire dataset or mitigate the impact of acquiring incomplete data [51]. It is necessary to find a trade-off between having an optimal temporal sequence of spaced-out distribution of gradient orientations and having a relatively fast acquisition. The specific scenarios must be determined a priori to select more effectively the subsets in post processing. Our findings indicate that using an optimization scheme for selecting (and ordering) gradient directions performs better than random, with no clear differences between the two subsampling methods tested. It is possible that this choice must be made while taking further data analysis processes into account. Therefore, when planning a novel research design (e.g., multi-session studies) it is crucial to take into account numerous significant factors beyond the chosen experimental protocol. Here we investigated the impact on: (1) different DKI parameters (FA, MD, MK, AD, AK, RD, and RK); and (2) different distribution characteristics (median, peak value, peak height, and peak width). Other forms of analysis (e.g., roi-based, voxelwise) may need a different number of volumes to obtain robust estimates and so should also be investigated in future research.

The generalisability of these results is subject to certain limitations. For instance, the selection of only female volunteers for this study resulted from the fact that the data was collected as part of a larger ongoing project that aimed to investigate white matter structure changes in female menstrual migraine patients when compared to gender-matched healthy controls. Although an interaction between sex and subsampling is not anticipated, future studies could evaluate a less homogeneous sample. Moreover, in this study, we only examined diffusion data from a typical DKI acquisition schemes of two-shells (b-values of 1000 and 2000 s/mm^2^) [20, 21]; however, in future studies we expect that our results could be generalized to other DKI schemes with different set of b-values. For instance, in future work, in addition to higher b-value, it could be useful to also consider lower b-values that better captures the effects of free-water in the brain [52]. Another factor that was not considered in this study is the number of bins used to compute the histogram (for example, using less bins to compute the histograms might result in more stable metrics to subsampling, however, it might be insufficient to capture enough information to differentiate between groups). One other aspect that should also be addressed in future is to obtain in vivo data from a sampling scheme that was originally produced using SC and compare both subsampling strategies, as the fact that we started from a sampling scheme optimized using EEM is likely to impact the performance of the SC method. On the other hand, our study can also be expanded to other diffusion models beyond DKI. The inherent complexity of the model in any approach requires the collection of a sufficient number of diffusion-weighted volumes to ensure that sufficient information is obtained for fitting the model [14].

## Conclusion

In this study, we evaluated the effects of data subsampling in DKI parameters and respective metrics extracted from histogram-based analysis. By comparing three different subsampling strategies (Opt_EEM_; Opt_SC_; and Random_TRUNC_), we demonstrated that the DKI estimates obtained from optimized subsampling strategies (Opt_EEM_ and Opt_SC_) were less susceptible to errors in comparison to the alternative non-optimized scheme (Random_TRUNC_). Overall, the subsampling showed greater effect on kurtosis parameters compared to diffusion parameters and the degree of bias showed to depend not only by the subsampling percentage and subsampling method but also on the data SNR. Although our simulations demonstrated that kurtosis parameters are more susceptible to biases than diffusion parameters, the *in vivo* findings indicate that most of the histogram characteristics for kurtosis parameters remain relatively stable even with data subsampling of up to 10–20% when optimal subsampling methods are employed. Consequently, this study suggests the suitability of using acquisition schemes with optimal acquisition ordering for DKI reconstruction and employing histogram-based analysis in studies where data acquisitions may be susceptible to scan interruptions.

## Supplementary Information

Below is the link to the electronic supplementary material.

Supplementary file1 (DOCX 3395 KB)

## Data Availability

The data that supports the findings of this study are available from the corresponding author, upon reasonable request.

## References

[1] Pierpaoli C, Basser PJ (1996) Toward a quantitative assessment of diffusion anisotropy. Magn Reson Med 36:893–906. 10.1002/mrm.1910360612 8946355 10.1002/mrm.1910360612

[2] Le Bihan D, Johansen-Berg H (2012) Diffusion MRI at 25: Exploring brain tissue structure and function. Neuroimage. 10.1016/j.neuroimage.2011.11.006 10.1016/j.neuroimage.2011.11.006 PMC3683822 22120012

[3] Soares JM, Marques P, Alves V, Sousa N (2013) A hitchhiker’s guide to diffusion tensor imaging. Front Neurosci. 10.3389/fnins.2013.00031 10.3389/fnins.2013.00031 PMC3594764 23486659

[4] Tournier J-D, Mori S, Leemans A (2011) Diffusion tensor imaging and beyond. Magn Reson Med 65:1532–56. 10.1002/mrm.22924 21469191 10.1002/mrm.22924 PMC3366862

[5] Jensen JH, Helpern JA, Ramani A, Lu H, Kaczynski K (2005) Diffusional kurtosis imaging: The quantification of non-Gaussian water diffusion by means of magnetic resonance imaging. Magn Reson Med 53:1432–40. 10.1002/mrm.20508 15906300 10.1002/mrm.20508

[6] Raab P, Hattingen E, Franz K, Zanella FE, Lanfermann H (2010) Cerebral gliomas: diffusional kurtosis imaging analysis of microstructural differences. Radiology. 10.1148/radiol.09090819 10.1148/radiol.09090819 20089718

[7] Hui ES, Fieremans E, Jensen JH, Tabesh A, Feng W, Bonilha L et al (2012) Stroke assessment with diffusional kurtosis imaging. Stroke. 10.1161/STROKEAHA.112.657742 10.1161/STROKEAHA.112.657742 PMC3479373 22933581

[8] Falangola MF, Jensen JH, Babb JS, Hu C, Castellanos FX, Di Martino A et al (2008) Age-related non-Gaussian diffusion patterns in the prefrontal brain. J Magn Reson Imag. 10.1002/jmri.21604 10.1002/jmri.21604 PMC2669671 19025941

[9] Ito K, Kudo M, Sasaki M, Saito A, Yamashita F, Harada T et al (2016) Detection of changes in the periaqueductal gray matter of patients with episodic migraine using quantitative diffusion kurtosis imaging: preliminary findings. Neuroradiology 58:115–20. 10.1007/s00234-015-1603-8 26446146 10.1007/s00234-015-1603-8

[10] Umesh Rudrapatna S, Wieloch T, Beirup K, Ruscher K, Mol W, Yanev P et al (2014) Can diffusion kurtosis imaging improve the sensitivity and specificity of detecting microstructural alterations in brain tissue chronically after experimental stroke? Comparisons with diffusion tensor imaging and histology. Neuroimage. 10.1016/j.neuroimage.2014.04.013 10.1016/j.neuroimage.2014.04.013 24742916

[11] Jensen JH, Helpern JA (2010) MRI quantification of non-Gaussian water diffusion by kurtosis analysis. NMR Biomed 23:698–710. 10.1002/nbm.1518 20632416 10.1002/nbm.1518 PMC2997680

[12] Hansen B, Lund TE, Sangill R, Jespersen SN (2013) Experimentally and computationally fast method for estimation of a mean kurtosis. Magn Reson Med. 10.1002/mrm.24743 10.1002/mrm.24743 23589312

[13] Dubois J, Poupon C, Lethimonnier F, Le Bihan D (2006) Optimized diffusion gradient orientation schemes for corrupted clinical DTI data sets. Magn Reson Mater Phys, Biol Med 19:134–43. 10.1007/s10334-006-0036-0 10.1007/s10334-006-0036-0 16896887

[14] Hutchinson EB, Avram AV, Irfanoglu MO, Koay CG, Barnett AS, Komlosh ME et al (2017) Analysis of the effects of noise, DWI sampling, and value of assumed parameters in diffusion MRI models. Magn Reson Med 78:1767–80. 10.1002/mrm.26575 28090658 10.1002/mrm.26575 PMC6084345

[15] Jones DK (2004) The Effect of Gradient Sampling Schemes on Measures Derived from Diffusion Tensor MRI: A Monte Carlo Study. Magn Reson Med 51:807–15. 10.1002/mrm.20033 15065255 10.1002/mrm.20033

[16] Papadakis NG, Murrills CD, Hall LD, Huang CLH, Adrian Carpenter T (2000) Minimal gradient encoding for robust estimation of diffusion anisotropy. Magn Reson Imaging. 10.1016/S0730-725X(00)00151-X 10.1016/s0730-725x(00)00151-x 10930776

[17] Poot DHJ, Den Dekker AJ, Achten E, Verhoye M, Sijbers J (2010) Optimal experimental design for diffusion kurtosis imaging. IEEE Trans Med Imag. 10.1109/TMI.2009.2037915 10.1109/TMI.2009.2037915 20199917

[18] Alexander DC (2008) A general framework for experiment design in diffusion MRI and its application in measuring direct tissue-microstructure features. Magn Reson Med. 10.1002/mrm.21646 10.1002/mrm.21646 18666109

[19] Caruyer E, Lenglet C, Sapiro G, Deriche R (2013) Design of multishell sampling schemes with uniform coverage in diffusion MRI. Magn Reson Med 69:1534–40. 10.1002/mrm.24736 23625329 10.1002/mrm.24736 PMC5381389

[20] Yokosawa S, Sasaki M, Bito Y, Ito K, Yamashita F, Goodwin J et al (2016) Optimization of scan parameters to reduce acquisition time for diffusion kurtosis imaging at 1.5T. Magn Reson Med Sci. 10.2463/mrms.2014-0139 10.2463/mrms.2014-0139 26104078

[21] Fukunaga I, Hori M, Masutani Y, Hamasaki N, Sato S, Suzuki Y et al (2013) Effects of diffusional kurtosis imaging parameters on diffusion quantification. Radiol Phys Technol. 10.1007/s12194-013-0206-5 10.1007/s12194-013-0206-5 PMC3709076 23536232

[22] Veraart J, Poot DHJ, Van Hecke W, Blockx I, Van der Linden A, Verhoye M et al (2011) More accurate estimation of diffusion tensor parameters using diffusion kurtosis imaging. Magn Reson Med : Off J Soc Magn Reson Med/Soc Magn Reson Med 65:138–45. 10.1002/mrm.22603 10.1002/mrm.22603 20878760

[23] Tabesh A, Jensen JH, Ardekani BA, Helpern JA (2011) Estimation of tensors and tensor-derived measures in diffusional kurtosis imaging. Magn Reson Med. 10.1002/mrm.22655 10.1002/mrm.22655 PMC3042509 21337412

[24] Deriche R, Calder J, Descoteaux M (2009) Optimal real-time Q-ball imaging using regularized Kalman filtering with incremental orientation sets. Med Image Anal. 10.1016/j.media.2009.05.008 10.1016/j.media.2009.05.008 19586794

[25] Cook PA, Symms M, Boulby PA, Alexander DC (2007) Optimal acquisition orders of diffusion-weighted MRI measurements. J Magn Reson Imag. 10.1002/jmri.20905 10.1002/jmri.20905 17457801

[26] Cheng J, Shen D, Yap P-T, Basser PJ (2018) Single- and multiple-shell uniform sampling schemes for diffusion MRI using spherical codes. IEEE Trans Med Imag 37:185–99. 10.1109/TMI.2017.2756072 10.1109/TMI.2017.2756072 PMC5867228 28952937

[27] Tournier JD, Smith R, Raffelt D, Tabbara R, Dhollander T, Pietsch M et al (2019) MRtrix3: A fast, flexible and open software framework for medical image processing and visualisation. Neuroimage. 10.1016/j.neuroimage.2019.116137 10.1016/j.neuroimage.2019.116137 31473352

[28] Jones DK, Horsfield MA, Simmons A (1999) Optimal strategies for measuring diffusion in anisotropic systems by magnetic resonance imaging. Magn Reson Med 42:515–25 10467296

[29] Cheng J, Shen D, Yap P-T (2014) Designing single- and multiple-shell sampling schemes for diffusion MRI using spherical code Lecture Notes in Computer Science (including subseries. Lecture Notes in Artificial Intelligence and Lecture Notes in Bioinformatics). LNCS 8675:281–8. 10.1007/978-3-319-10443-0_36 10.1007/978-3-319-10443-0_36 PMC8167438 25320810

[30] Henriques RN, Correia MM, Nunes RG, Ferreira HA (2015) Exploring the 3D geometry of the diffusion kurtosis tensor-Impact on the development of robust tractography procedures and novel biomarkers. Neuroimage 111:85–99. 10.1016/j.neuroimage.2015.02.004 25676915 10.1016/j.neuroimage.2015.02.004

[31] Henriques RN, Correia MM, Marrale M, Huber E, Kruper J, Koudoro S et al (2021) Diffusional kurtosis imaging in the diffusion imaging in Python Project. Front Hum Neurosci. 10.3389/fnhum.2021.675433 10.3389/fnhum.2021.675433 PMC8327208 34349631

[32] Garyfallidis E, Brett M, Amirbekian B, Rokem A, van der Walt S, Descoteaux M et al (2014) Dipy, a library for the analysis of diffusion MRI data. Front Neuroinform. 10.3389/fninf.2014.00008 10.3389/fninf.2014.00008 PMC3931231 24600385

[33] Ades-Aron B, Veraart J, Kochunov P, McGuire S, Sherman P, Kellner E et al (2018) Evaluation of the accuracy and precision of the diffusion parameter EStImation with Gibbs and NoisE removal pipeline. Neuroimage 183:532–43. 10.1016/j.neuroimage.2018.07.066 30077743 10.1016/j.neuroimage.2018.07.066 PMC6371781

[34] Veraart J, Novikov DS, Christiaens D, Ades-aron B, Sijbers J, Fieremans E (2016) Denoising of diffusion MRI using random matrix theory. Neuroimage 142:394–406. 10.1016/j.neuroimage.2016.08.016 27523449 10.1016/j.neuroimage.2016.08.016 PMC5159209

[35] Kellner E, Dhital B, Kiselev VG, Reisert M (2016) Gibbs-ringing artifact removal based on local subvoxel-shifts. Magn Reson Med. 10.1002/mrm.26054 10.1002/mrm.26054 26745823

[36] Koay CG, Basser PJ (2006) Analytically exact correction scheme for signal extraction from noisy magnitude MR signals. J Magn Reson 179:317–22. 10.1016/j.jmr.2006.01.016 16488635 10.1016/j.jmr.2006.01.016

[37] Andersson JLR, Skare S, Ashburner J (2003) How to correct susceptibility distortions in spin-echo echo-planar images: Application to diffusion tensor imaging. Neuroimage 20:870–88. 10.1016/S1053-8119(03)00336-7 14568458 10.1016/S1053-8119(03)00336-7

[38] Andersson JLR, Sotiropoulos SN (2016) An integrated approach to correction for off-resonance effects and subject movement in diffusion MR imaging. Neuroimage 125:1063–78. 10.1016/j.neuroimage.2015.10.019 26481672 10.1016/j.neuroimage.2015.10.019 PMC4692656

[39] Andersson JLR, Sotiropoulos SN (2015) Non-parametric representation and prediction of single- and multi-shell diffusion-weighted MRI data using Gaussian processes. Neuroimage 122:166–76. 10.1016/j.neuroimage.2015.07.067 26236030 10.1016/j.neuroimage.2015.07.067 PMC4627362

[40] Smith SM, Jenkinson M, Johansen-Berg H, Rueckert D, Nichols TE, Mackay CE et al (2006) Tract-based spatial statistics: Voxelwise analysis of multi-subject diffusion data. Neuroimage 31:1487–505 16624579 10.1016/j.neuroimage.2006.02.024

[41] Fouto AR, Nunes RG, Pinto J, Alves L, Calado S, Gonçalves C et al (2022) Impact of white-matter mask selection on DTI histogram-based metrics as potential biomarkers in cerebral small vessel disease. Magn Reson Mater Phys, Biol Med. 10.1007/s10334-021-00991-4 10.1007/s10334-021-00991-4 34997895

[42] Baykara E, Gesierich B, Adam R, Tuladhar AM, Biesbroek JM, Koek HL et al (2016) A Novel Imaging Marker for Small Vessel Disease Based on Skeletonization of White Matter Tracts and Diffusion Histograms. Ann Neurol 80:581–92. 10.1002/ana.24758 27518166 10.1002/ana.24758

[43] Helpern JA, Adisetiyo V, Falangola MF, Hu C, Di Martino A, Williams K et al (2011) Preliminary evidence of altered gray and white matter microstructural development in the frontal lobe of adolescents with attention-deficit hyperactivity disorder: A diffusional kurtosis imaging study. J Magn Reson Imag. 10.1002/jmri.22397 10.1002/jmri.22397 PMC3492944 21182116

[44] Gard A, Al-Husseini A, Kornaropoulos EN, De Maio A, Tegner Y, Björkman-Burtscher I et al (2022) Post-Concussive Vestibular Dysfunction Is Related to Injury to the Inferior Vestibular Nerve. J Neurotrauma. 10.1089/neu.2021.0447 10.1089/neu.2021.0447 PMC9225415 35171721

[45] Zhuo J, Xu S, Proctor JL, Mullins RJ, Simon JZ, Fiskum G et al (2012) Diffusion kurtosis as an in vivo imaging marker for reactive astrogliosis in traumatic brain injury. Neuroimage. 10.1016/j.neuroimage.2011.07.050 10.1016/j.neuroimage.2011.07.050 PMC3614502 21835250

[46] Henriques RN, Henson R, Correia MM (2023) Unique information from common diffusion MRI models about white-matter differences across the human adult lifespan. Imag Neurosci 1:1–25. 10.1162/imag_a_00051 10.1162/imag_a_00051 PMC12327084 40799710

[47] Jensen JH, Falangola MF, Hu C, Tabesh A, Rapalino O, Lo C et al (2011) Preliminary observations of increased diffusional kurtosis in human brain following recent cerebral infarction. NMR Biomed. 10.1002/nbm.1610 10.1002/nbm.1610 PMC3549661 20960579

[48] Hui ES, Fieremans E, Jensen JH et al (2012) Stroke assessment with diffusional kurtosis imaging. Stroke. 10.1161/STROKEAHA.112.657742 10.1161/STROKEAHA.112.657742 PMC3479373 22933581

[49] Henriques RN, Jespersen SN, Jones DK, Veraart J (2021) Toward more robust and reproducible diffusion kurtosis imaging. Magn Reson Med. 10.1002/mrm.28730 10.1002/mrm.28730 PMC8199974 33829542

[50] Kornaropoulos EN, Winzeck S, Rumetshofer T, Wikstrom A, Knutsson L, Correia MM et al (2022) Sensitivity of Diffusion MRI to White Matter Pathology: Influence of Diffusion Protocol, Magnetic Field Strength, and Processing Pipeline in Systemic Lupus Erythematosus. Front Neurol. 10.3389/fneur.2022.837385 10.3389/fneur.2022.837385 PMC9087851 35557624

[51] Dubois J, Poupon C, Lethimonnier F, Le Bihan D (2006) Optimized diffusion gradient orientation schemes for corrupted clinical DTI data sets. Magn Reson Mater Phys, Biol Med 19:134–43. 10.1007/s10334-006-0036-0 10.1007/s10334-006-0036-0 16896887

[52] Pierpaoli C, Jones DK (2004) Removing CSF Contamination in Brain DT-MRIs by Using a Two-Compartment Tensor Model. Proc. Intl. Soc. Mag. Reson. Med 11:1215

