# Supplementary material for: Impact of truncating diffusion MRI scans on diffusional kurtosis imaging
Source: MAGMA. 2024 Feb 23;37(5):859–72. doi: 10.1007/s10334-024-01153-y (PMC11452422; doi:10.1007/s10334-024-01153-y)
Supplement: Supplementary file 1 — Supplementary file1 (DOCX 3395 KB) [file 10334_2024_1153_MOESM1_ESM.docx]

1. **Supplementary Material**

**Figure S1** – Relative difference (in percentage) from the ground truth (full) for FA, MD, AD, and RD using the following methods: Opt_EEM_ (in red), Opt_SC_ (in blue), and Random_TRUNC_ (in green).


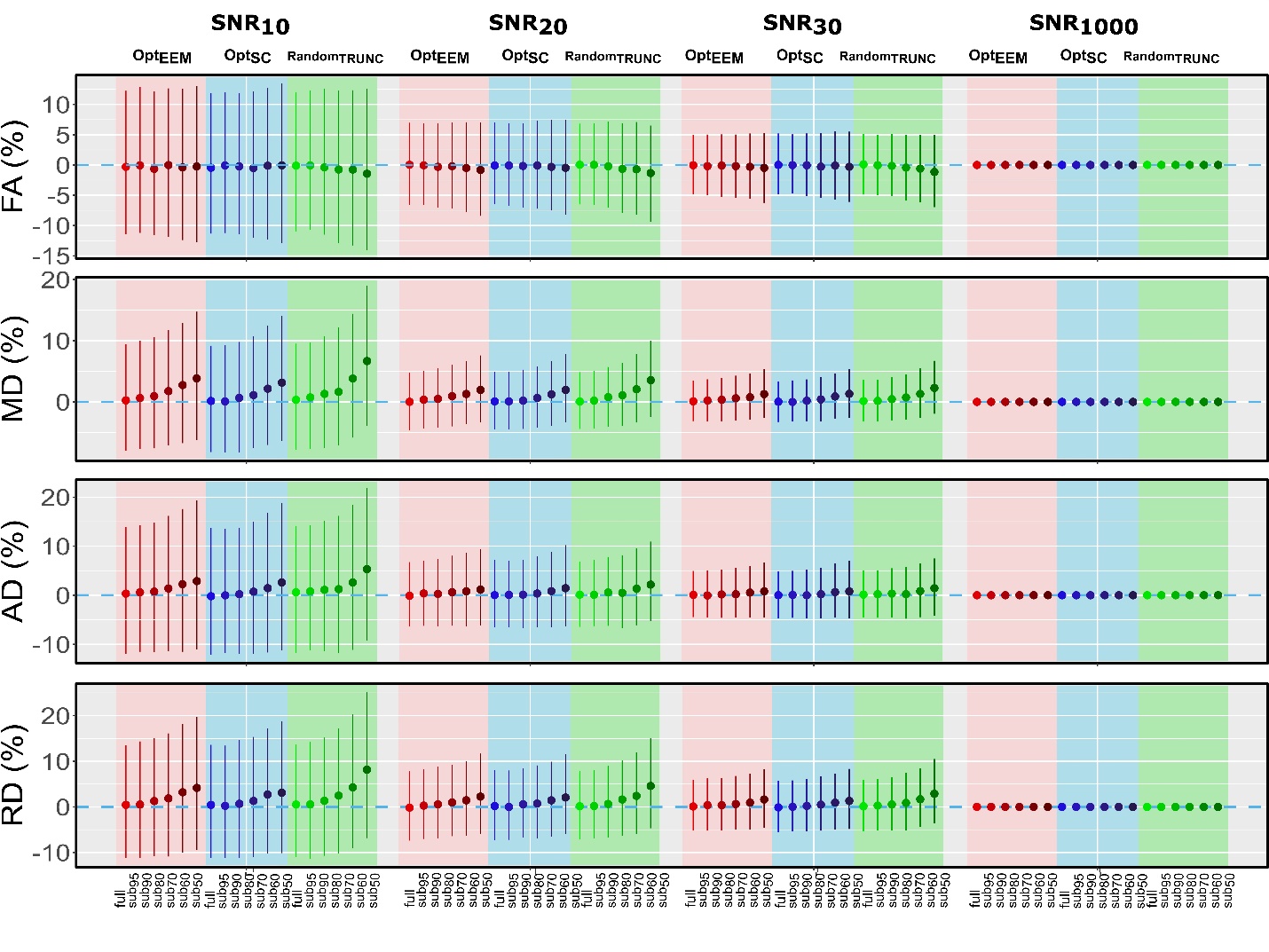


**Figure S2** – Relative difference (in percentage) from the ground truth (full) for MK, AK and RK using the following methods: Opt_EEM_ (in red), Opt_SC_ (in blue), and Random_TRUNC_ (in green).


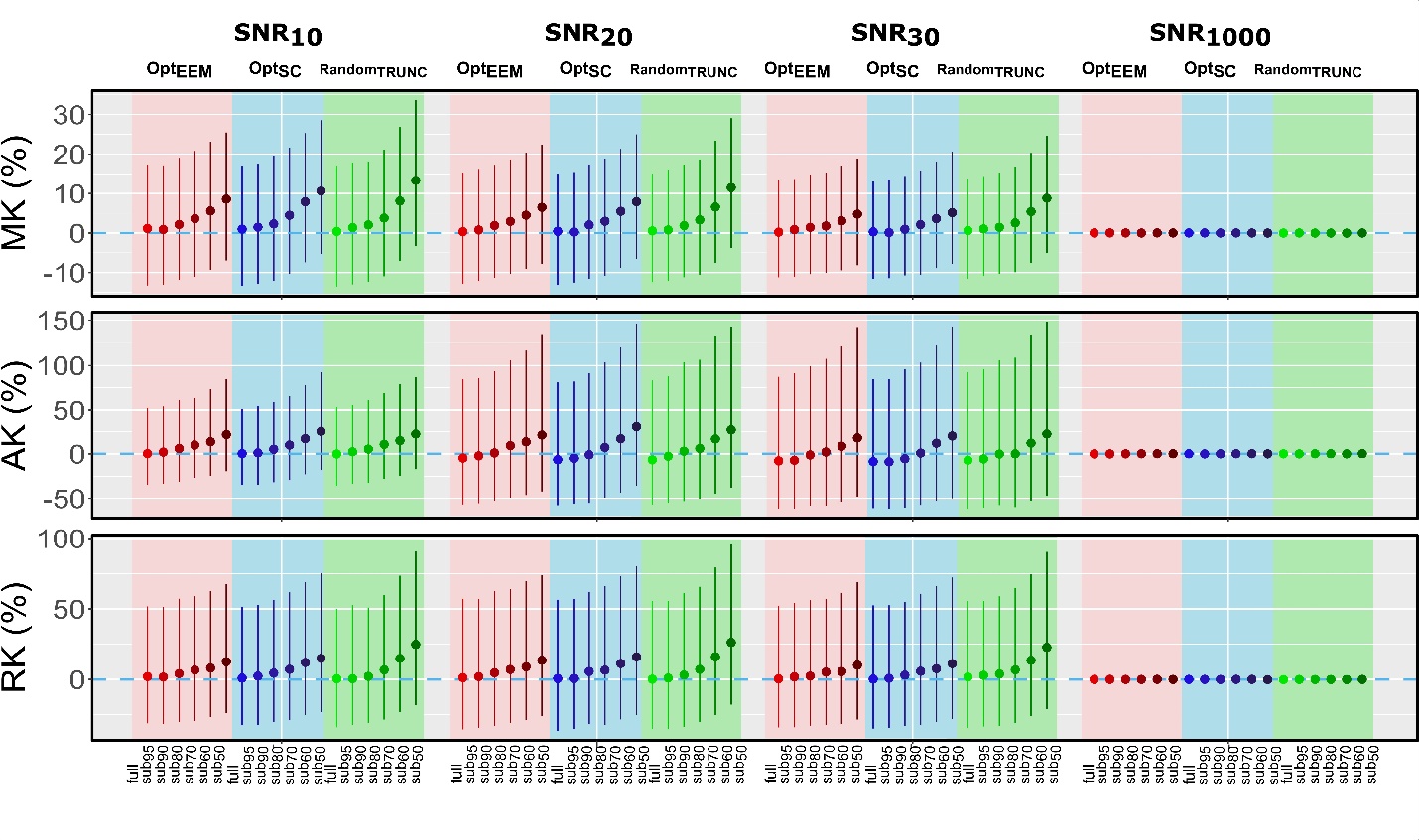


**Figure S3** – Illustrative examples of overlapping whole-brain histograms derived from fully and subsampled FA maps using both methods: Opt_EEM_ (in red) and Opt_SC_ (in blue); black lines highlight the histogram-based metrics (median and peak width). Each row corresponds to a different subject.


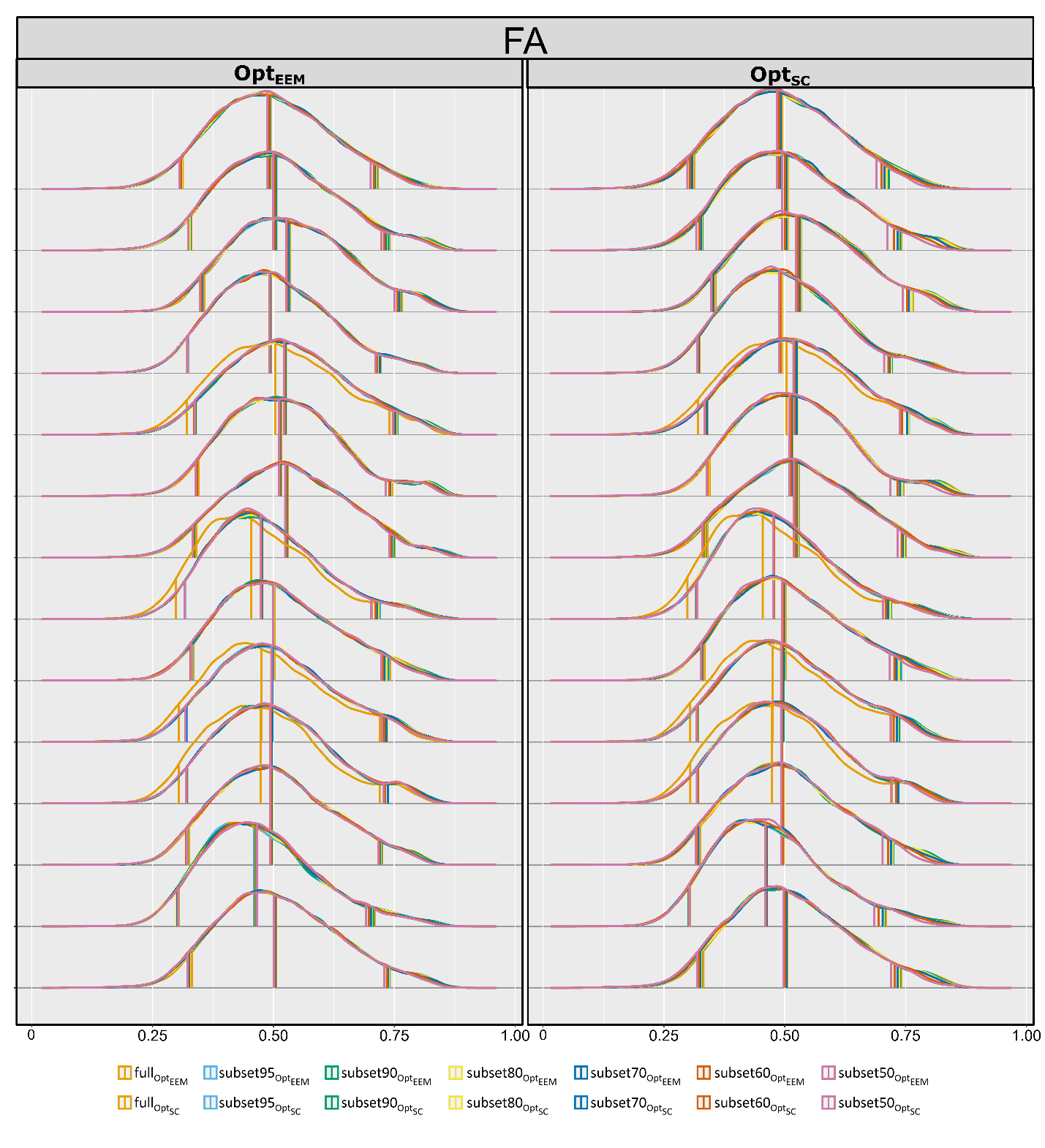


**Figure S4** – Illustrative examples of overlapping whole-brain histograms derived from fully and subsampled MD maps (in mm^2^s^-1^) using both methods: Opt_EEM_ (in red) and Opt_SC_ (in blue); black lines highlight the histogram-based metrics (median and peak width). Each row corresponds to a different subject.


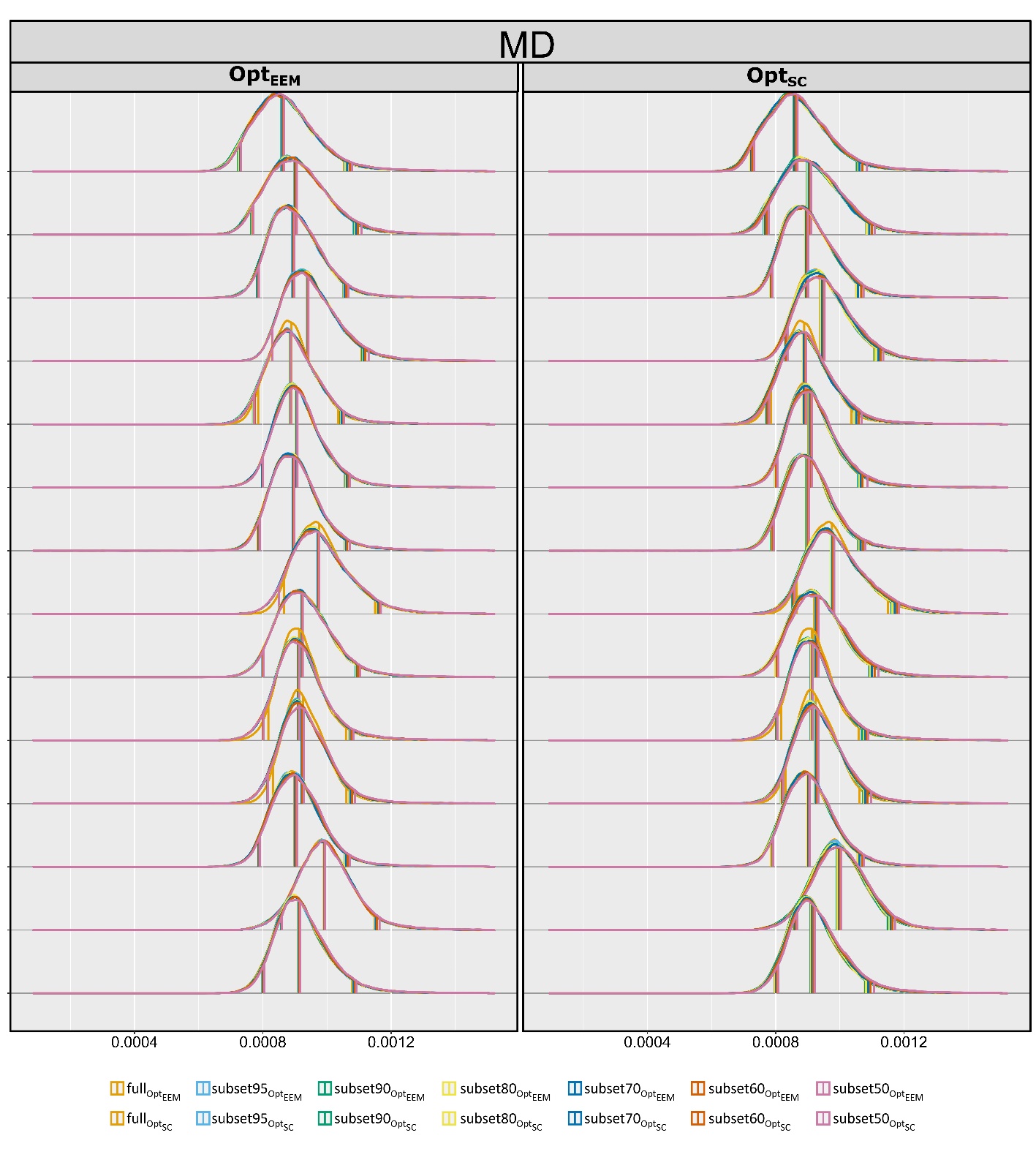


**Figure S5** – Illustrative examples of overlapping whole-brain histograms derived from fully and subsampled AD maps (in mm^2^s^-1^) using both methods: Opt_EEM_ (in red) and Opt_SC_ (in blue); black lines highlight the histogram-based metrics (median and peak width). Each row corresponds to a different subject.


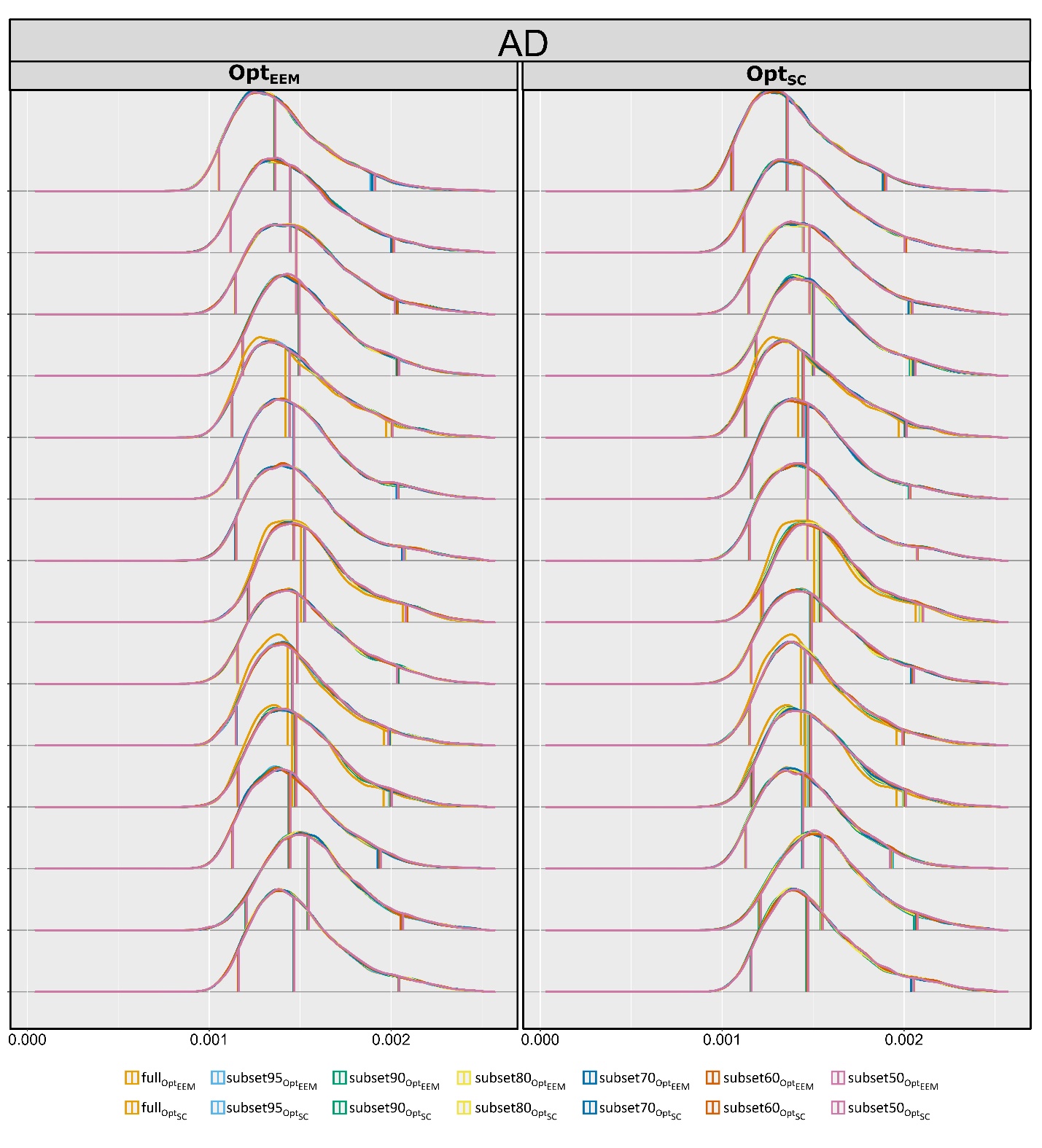


**Figure S6** – Illustrative examples of overlapping whole-brain histograms derived from fully and subsampled RD maps (in mm^2^s^-1^) using both methods: Opt_EEM_ (in red) and Opt_SC_ (in blue); black lines highlight the histogram-based metrics (median and peak width). Each row corresponds to a different subject.


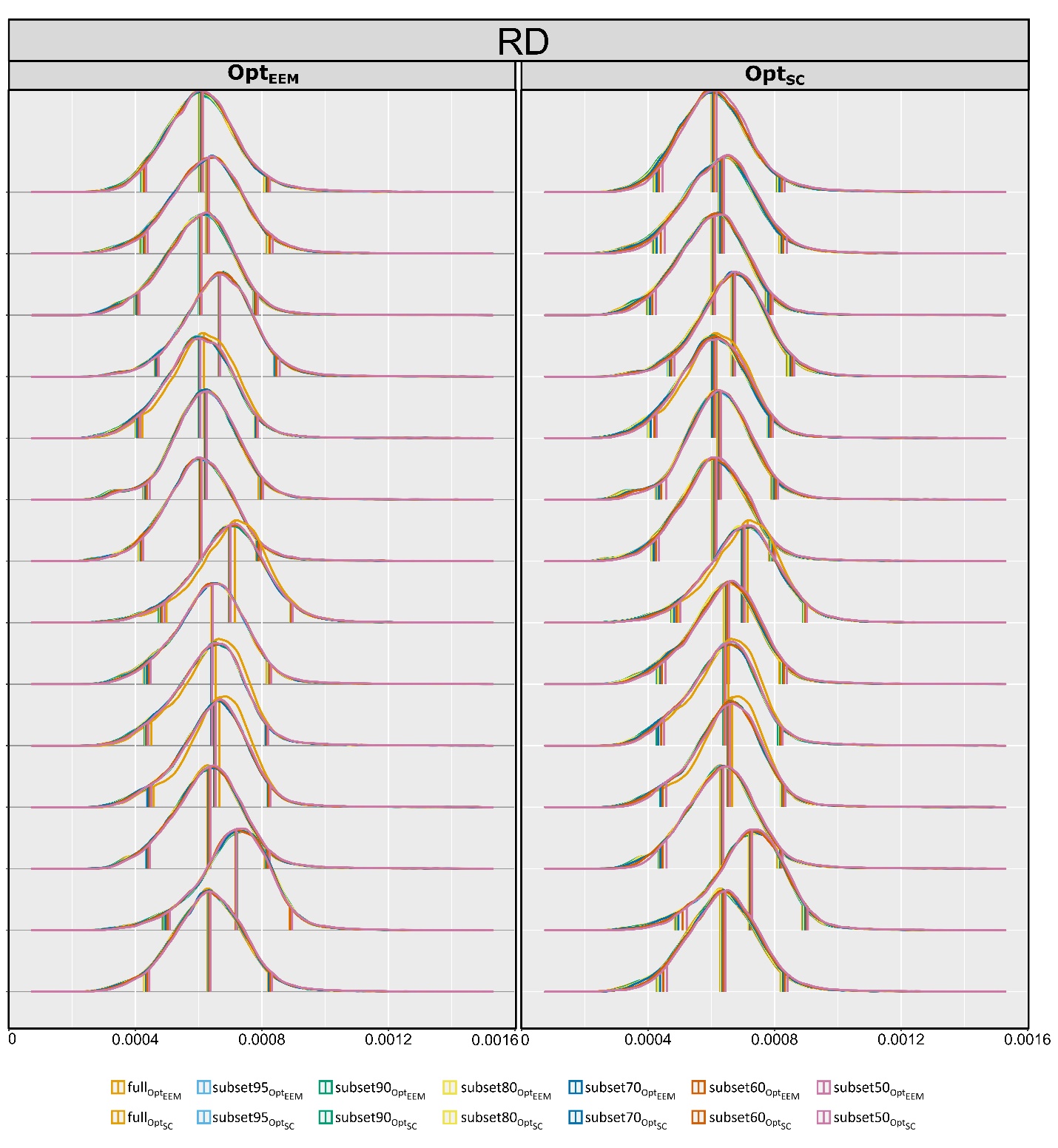


**Figure S7** – Illustrative examples of overlapping whole-brain histograms derived from fully and subsampled MK maps using both methods: Opt_EEM_ (in red) and Opt_SC_ (in blue); black lines highlight the histogram-based metrics (median and peak width). Each row corresponds to a different subject.


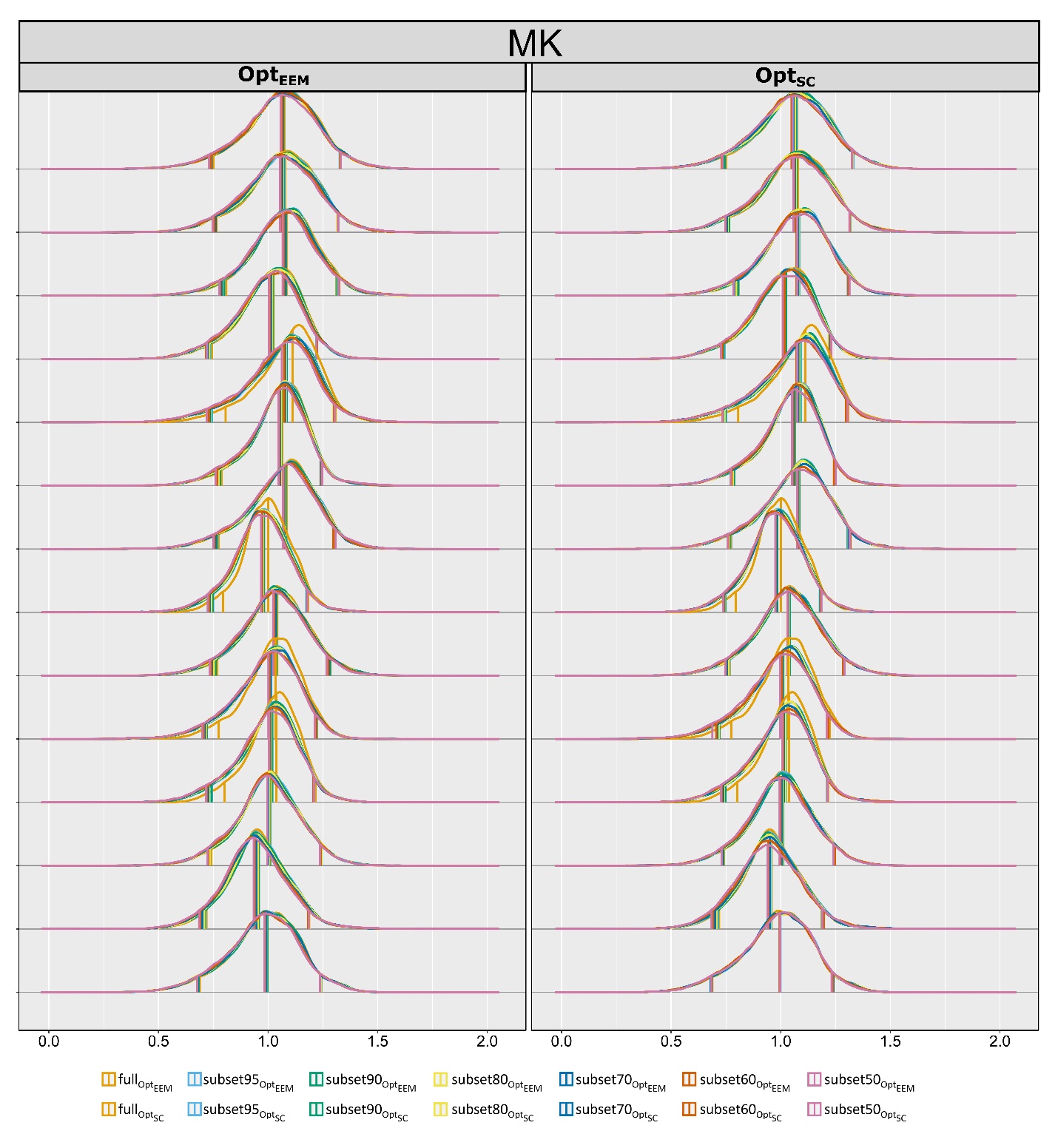


**Figure S8** – Illustrative examples of overlapping whole-brain histograms derived from fully and subsampled AK maps using both methods: Opt_EEM_ (in red) and Opt_SC_ (in blue); black lines highlight the histogram-based metrics (median and peak width). Each row corresponds to a different subject.


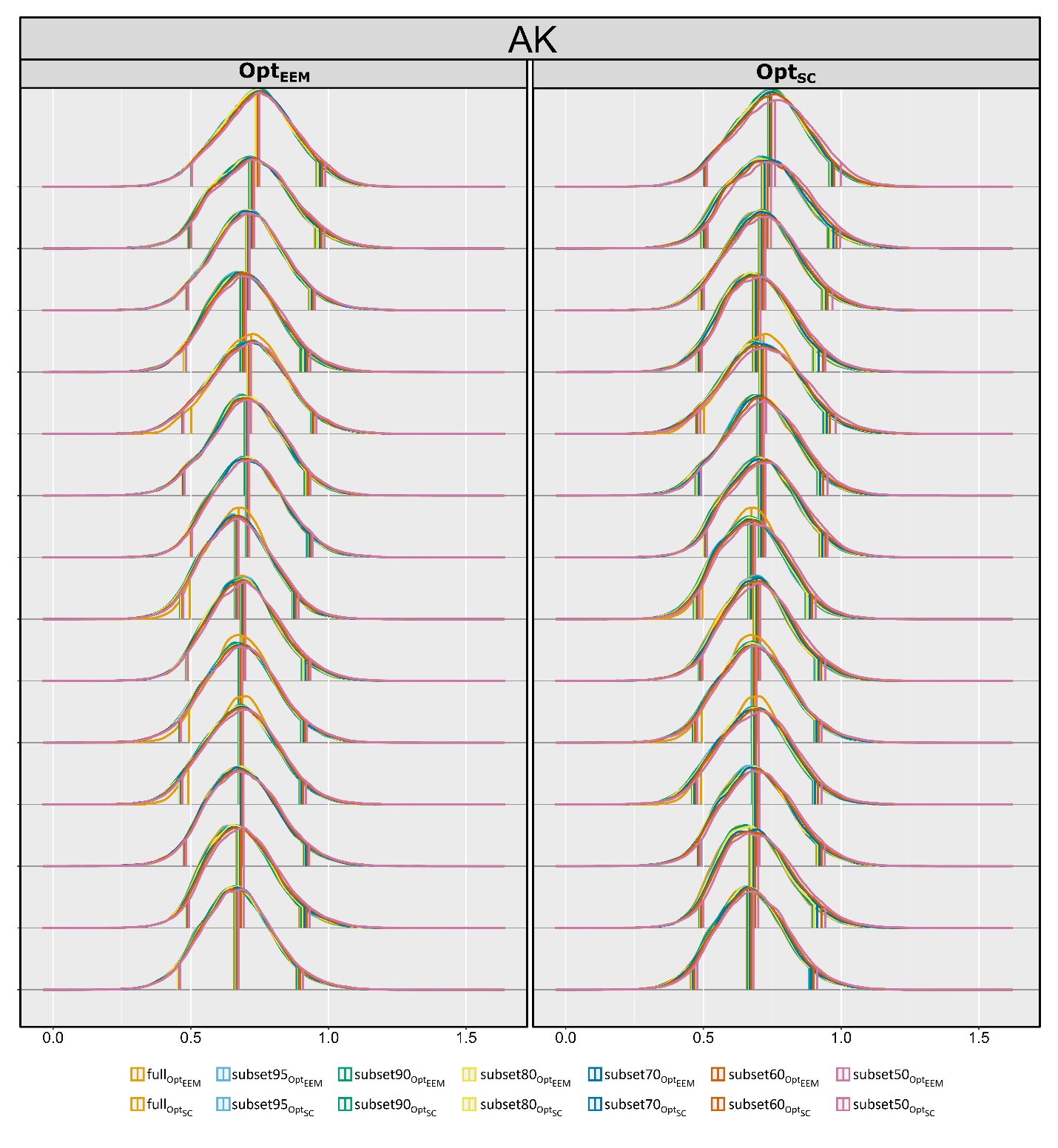


**Figure S9** – Relative difference (in percentage) from the ground truth (full): Opt_EEM_ (in red) and Opt_SC_ (in blue) for each histogram DTI metric extracted from the skeletonized parametric map estimated from the in vivo brain data.


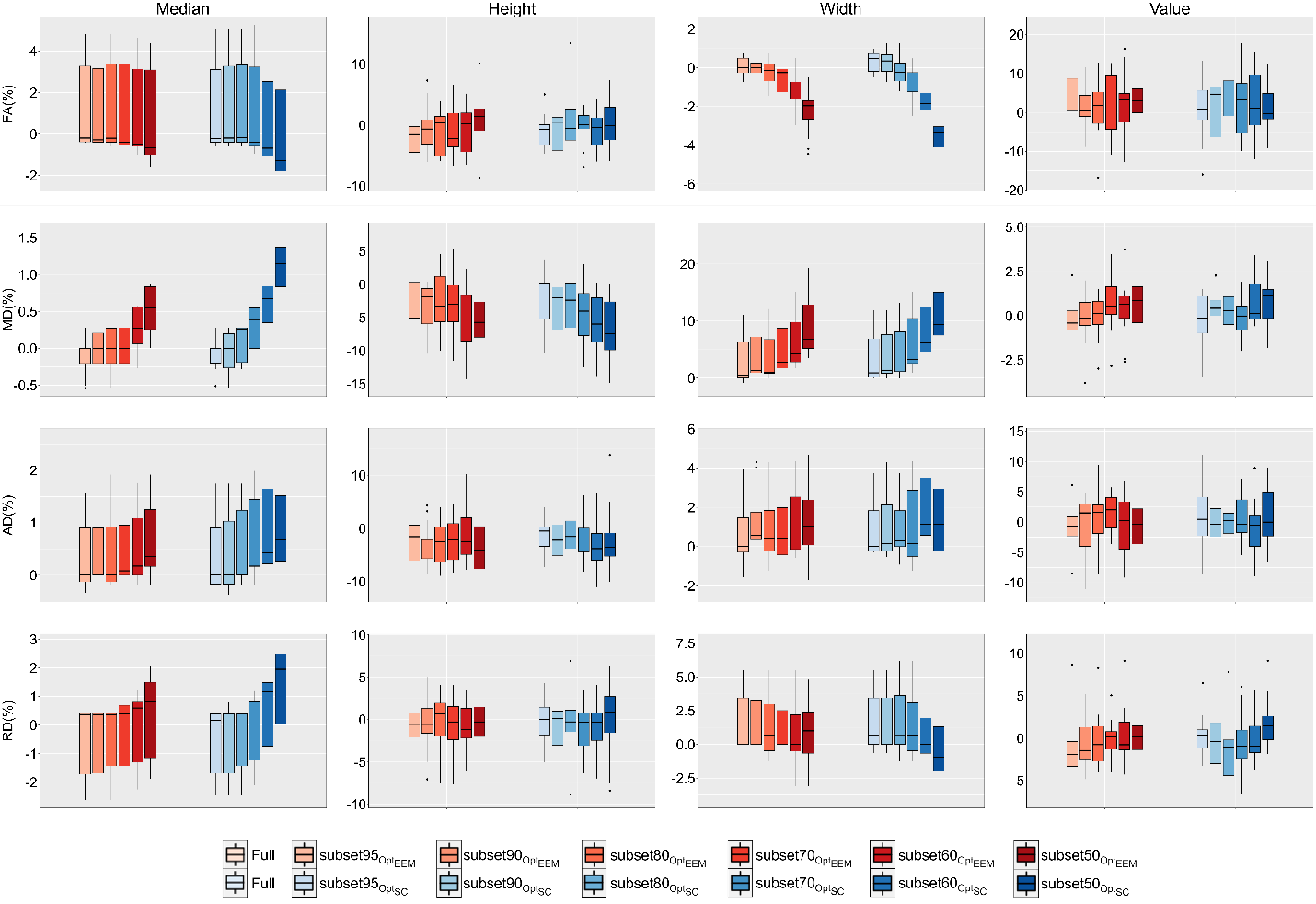


**Figure S10** – Relative difference (in percentage) from the ground truth (full): Opt_EEM_ (in red) and Opt_SC_ (in blue) for each histogram DKI metric extracted from the skeletonized parametric map estimated from the in vivo brain data.


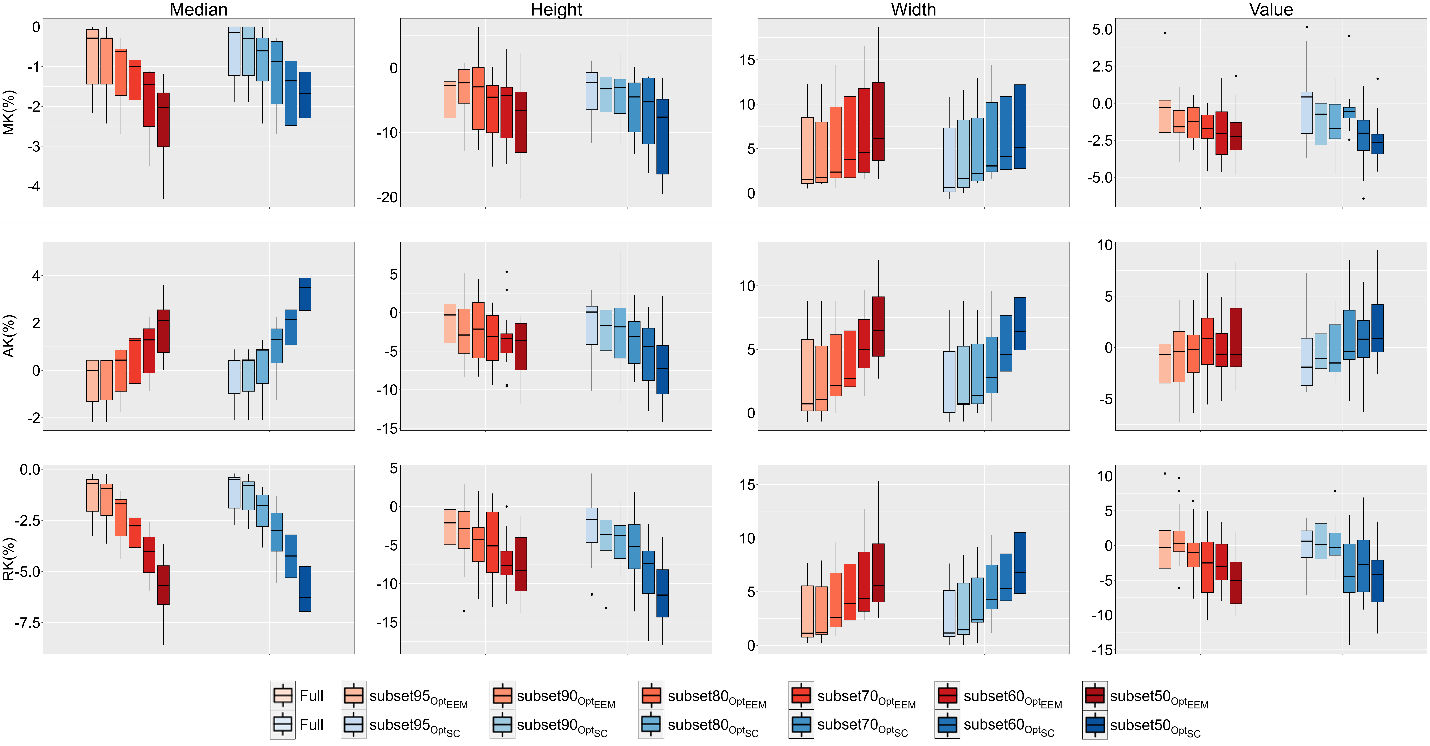


**Table S1** – **Summary of the median and interquartile (IQR) ranges of the relative difference error (in percentage) of FA, MD, AD, RD, MK, AK and RK for all subsampling methods (Opt_EEM_, Opt_SC_, Random_TRUNC_) for a typical SNR of 20.**

| **Map** | **Subsampling** | **Median** | | | **IQR** | | |
| --- | --- | --- | --- | --- | --- | --- | --- |
|  |  | Opt_EEM_ | Opt_SC_ | Random_TRUNC_ | Opt_EEM_ | Opt_SC_ | Random_TRUNC_ |
| **FA** | subset95 | -0.026 | 0.071 | -0.035 | 13.53 | 13.50 | 13.34 |
|  | subset90 | 0.037 | 0.086 | -0.024 | 13.47 | 13.62 | 13.50 |
|  | subset80 | 0.30 | 0.16 | 0.21 | 13.94 | 13.91 | 14.15 |
|  | subset70 | 0.19 | 0.085 | 0.64 | 14.21 | 14.55 | 14.82 |
|  | subset60 | 0.48 | 0.31 | 0.72 | 14.90 | 14.99 | 15.34 |
|  | subset50 | 0.83 | 0.47 | 1.34 | 15.46 | 15.70 | 15.89 |
| **MD** | subset95 | -0.009 | -0.097 | -0.056 | 9.35 | 9.33 | 9.29 |
|  | subset90 | -0.35 | -0.10 | -0.20 | 9.47 | 9.35 | 9.42 |
|  | subset80 | -0.48 | -0.20 | -0.77 | 9.75 | 9.69 | 9.73 |
|  | subset70 | -0.92 | -0.63 | -1.08 | 10.03 | 10.03 | 10.35 |
|  | subset60 | -1.30 | -1.25 | -2.07 | 10.32 | 10.62 | 11.22 |
|  | subset50 | -1.95 | -1.96 | -3.55 | 10.97 | 11.17 | 12.46 |
| **AD** | subset95 | 0.11 | -0.012 | -0.12 | 13.16 | 13.73 | 13.36 |
|  | subset90 | -0.36 | -0.082 | -0.10 | 13.33 | 13.45 | 13.51 |
|  | subset80 | -0.29 | -0.12 | -0.56 | 13.72 | 13.89 | 14.08 |
|  | subset70 | -0.61 | -0.35 | -0.49 | 14.28 | 14.39 | 14.86 |
|  | subset60 | -1.34 | -0.85 | -0.81 | 15.65 | 15.46 | 14.98 |
|  | subset50 | -1.16 | -1.43 | -2.16 | 15.67 | 16.52 | 16.24 |
| **RD** | subset95 | 0.15 | -0.19 | -0.14 | 15.20 | 15.28 | 14.97 |
|  | subset90 | -0.28 | 0.015 | -0.18 | 15.25 | 15.19 | 14.97 |
|  | subset80 | -0.54 | -0.56 | -0.65 | 15.83 | 15.28 | 15.74 |
|  | subset70 | -0.98 | -0.72 | -1.62 | 16.60 | 15.66 | 16.38 |
|  | subset60 | -1.45 | -1.44 | -2.41 | 16.40 | 16.38 | 17.83 |
|  | subset50 | -2.25 | -2.06 | -4.59 | 17.55 | 17.43 | 19.80 |
| **MK** | subset95 | -0.32 | -0.42 | -0.53 | 28.17 | 28.15 | 27.47 |
|  | subset90 | -0.76 | -0.25 | -0.77 | 28.34 | 27.94 | 28.17 |
|  | subset80 | -1.92 | -2.05 | -1.88 | 28.62 | 28.93 | 28.55 |
|  | subset70 | -2.94 | -2.98 | -3.34 | 28.88 | 29.54 | 29.23 |
|  | subset60 | -4.51 | -5.50 | -6.60 | 29.45 | 30.13 | 30.92 |
|  | subset50 | -6.50 | -7.95 | -11.49 | 30.08 | 31.54 | 32.87 |
| **AK** | subset95 | 4.69 | 6.44 | 6.71 | 141.10 | 138.55 | 140.73 |
|  | subset90 | 2.27 | 4.97 | 2.61 | 140.91 | 137.54 | 142.93 |
|  | subset80 | -1.02 | 1.01 | -3.02 | 146.31 | 146.65 | 156.54 |
|  | subset70 | -9.16 | -7.25 | -6.06 | 154.79 | 152.61 | 156.74 |
|  | subset60 | -13.53 | -16.90 | -16.72 | 162.98 | 163.52 | 177.56 |
|  | subset50 | -21.11 | -30.54 | -27.03 | 176.50 | 182.20 | 180.44 |
| **RK** | subset95 | -1.29 | -0.58 | -0.16 | 92.66 | 93.15 | 90.45 |
|  | subset90 | -2.05 | -0.68 | -0.97 | 91.14 | 92.30 | 90.70 |
|  | subset80 | -4.79 | -5.65 | -3.10 | 95.50 | 93.97 | 94.68 |
|  | subset70 | -7.06 | -6.58 | -7.15 | 95.00 | 98.26 | 95.98 |
|  | subset60 | -8.97 | -11.31 | -16.11 | 98.26 | 101.61 | 105.26 |
|  | subset50 | -13.58 | -16.10 | -26.33 | 100.20 | 104.59 | 113.43 |

| **Map** | **Metric** | **Subsampling** | **Method** | **Subsampling*Method** |
| --- | --- | --- | --- | --- |
| **FA** | Median | **<0.001** | **<0.001** | **<0.001** |
|  | Peak height | 0.095 | 0.451 | 0.683 |
|  | Peak width | **<0.001** | **<0.001** | **<0.001** |
|  | Peak value | 0.371 | 0.777 | 0.057 |
| **MD** | Median | **<0.001** | **<0.001** | **<0.001** |
|  | Peak height | **<0.001** | **0.047** | 0.561 |
|  | Peak width | **<0.001** | **<0.001** | **<0.001** |
|  | Peak value | **0.020** | 0.605 | 0.326 |
| **AD** | Median | **<0.001** | **0.019** | **<0.001** |
|  | Peak height | **0.019** | 0.202 | **0.033** |
|  | Peak width | **<0.001** | 0.555 | 0.725 |
|  | Peak value | 0.497 | 0.461 | 0.117 |
| **RD** | Median | **<0.001** | **<0.001** | **<0.001** |
|  | Peak height | 0.542 | 0.981 | 0.944 |
|  | Peak width | **<0.001** | 0.632 | **<0.001** |
|  | Peak value | **0.044** | 0.310 | **0.028** |
| **MK** | Median | **<0.001** | **0.006** | **0.010** |
|  | Peak height | **<0.001** | 0.243 | 0.123 |
|  | Peak width | **<0.001** | 0.102 | 0.076 |
|  | Peak value | **<0.001** | **0.006** | **0.010** |
| **AK** | Median | **<0.001** | **<0.001** | **<0.001** |
|  | Peak height | **<0.001** | **0.045** | 0.158 |
|  | Peak width | **<0.001** | 0.157 | 0.342 |
|  | Peak value | **0.013** | 0.422 | 0.966 |
| **RK** | Median | **<0.001** | 0.362 | 0.160 |
|  | Peak height | **<0.001** | **0.026** | **0.007** |
|  | Peak width | **<0.001** | 0.218 | **0.015** |
|  | Peak value | **<0.001** | 0.921 | 0.882 |

**Table S2** – **Results of the** **2-way repeated measures ANOVA analysis are summarized.** The table shows that there are significant main effects for both Subsampling and Method on different histogram metrics for several diffusion parameters. There are also significant interactions (highlighted in blue) between Subsampling and Method, implying that the effects of Subsampling on each histogram metric vary depending on the Method of subsampling applied.

**Table S3** – **The median and IQR ranges of the relative error (in percentage) of the FA histogram metrics (median, peak height, peak width, and peak value) for all subsampling methods (Opt_EEM_, Opt_SC_).**

| **Map** | **Metric** | **Subsampling** | **Median** | | **IQR** | |
| --- | --- | --- | --- | --- | --- | --- |
|  |  |  | **Opt_EEM_** | **Opt_SC_** | **Opt_EEM_** | **Opt_SC_** |
| FA | Median | subset95 | -0.20 | -0.21 | 3.66 | 3.52 |
|  |  | subset90 | -0.29 | -0.20 | 3.56 | 3.68 |
|  |  | subset80 | -0.20 | -0.19 | 3.77 | 3.73 |
|  |  | subset70 | -0.39 | -0.40 | 3.91 | 3.83 |
|  |  | subset60 | -0.49 | -0.69 | 3.74 | 3.62 |
|  |  | subset50 | -0.67 | -1.28 | 4.10 | 3.93 |
|  | Peak Height | subset95 | -1.54 | -0.73 | 4.24 | 3.28 |
|  |  | subset90 | -0.63 | 0.46 | 4.01 | 5.43 |
|  |  | subset80 | 0.35 | -0.49 | 6.47 | 5.11 |
|  |  | subset70 | -2.18 | 0.10 | 5.52 | 2.17 |
|  |  | subset60 | 0.24 | -0.43 | 6.40 | 4.47 |
|  |  | subset50 | 1.43 | -0.05 | 3.62 | 5.36 |
|  | Peak Width | subset95 | 0 | 0.49 | 0.75 | 0.92 |
|  |  | subset90 | 0 | 0.36 | 0.50 | 0.86 |
|  |  | subset80 | -0.12 | -0.24 | 0.86 | 0.924 |
|  |  | subset70 | -0.25 | -0.99 | 1.19 | 1.00 |
|  |  | subset60 | -0.97 | -1.83 | 0.90 | 0.86 |
|  |  | subset50 | -1.96 | -3.32 | 0.97 | 1.06 |
|  | Peak Value | subset95 | 3.44 | 0.88 | 8.31 | 7.55 |
|  |  | subset90 | 0.44 | 4.75 | 5.45 | 12.98 |
|  |  | subset80 | 1.91 | 6.52 | 8.10 | 9.06 |
|  |  | subset70 | 3.52 | 3.20 | 13.71 | 12.86 |
|  |  | subset60 | 3.28 | 1.11 | 7.34 | 12.79 |
|  |  | subset50 | 2.96 | -0.30 | 6.17 | 6.60 |

**Table S4** – **The median and IQR ranges of the relative error (in percentage) of the MD histogram metrics (median, peak height, peak width, and peak value) for all subsampling methods (Opt_EEM_, Opt_SC_).**

| **Map** | **Metric** | **Subsampling** | **Median** | | **IQR** | |
| --- | --- | --- | --- | --- | --- | --- |
|  |  |  | **Opt_EEM_** | **Opt_SC_** | **Opt_EEM_** | **Opt_SC_** |
| MD | Median | subset95 | 0 | 0 | 0.21 | 0.20 |
|  |  | subset90 | 0 | 0 | 0.41 | 0.47 |
|  |  | subset80 | 0 | 0.26 | 0.48 | 0.47 |
|  |  | subset70 | 0 | 0.39 | 0.48 | 0.55 |
|  |  | subset60 | 0.27 | 0.67 | 0.49 | 0.49 |
|  |  | subset50 | 0.55 | 1.14 | 0.58 | 0.53 |
|  | Peak Height | subset95 | -1.78 | -1.73 | 5.37 | 5.50 |
|  |  | subset90 | -1.86 | -2.06 | 5.29 | 6.38 |
|  |  | subset80 | -3.24 | -2.37 | 6.82 | 6.72 |
|  |  | subset70 | -2.94 | -4.03 | 5.49 | 6.36 |
|  |  | subset60 | -3.38 | -5.97 | 7.01 | 6.73 |
|  |  | subset50 | -5.71 | -7.38 | 5.30 | 7.26 |
|  | Peak Width | subset95 | 0.46 | 0.91 | 6.32 | 6.68 |
|  |  | subset90 | 1.32 | 1.37 | 6.28 | 6.80 |
|  |  | subset80 | 0.94 | 2.32 | 5.92 | 6.95 |
|  |  | subset70 | 2.83 | 3.25 | 7.00 | 7.91 |
|  |  | subset60 | 4.23 | 6.18 | 7.00 | 7.82 |
|  |  | subset50 | 6.80 | 9.38 | 7.67 | 7.56 |
|  | Peak Value | subset95 | -0.42 | -0.14 | 2.10 | 2.10 |
|  |  | subset90 | -0.14 | 0.42 | 1.31 | 0.88 |
|  |  | subset80 | 0.14 | 0.29 | 1.31 | 1.58 |
|  |  | subset70 | 0.56 | -0.006 | 1.57 | 1.33 |
|  |  | subset60 | 0.67 | 0.13 | 1.27 | 1.98 |
|  |  | subset50 | 0.84 | 1.15 | 2.03 | 1.64 |

**Table S5** – **The median and IQR ranges of the relative error (in percentage) of the AD histogram metrics (median, peak height, peak width, and peak value) for all subsampling methods (Opt_EEM_, Opt_SC_).**

| **Map** | **Metric** | **Subsampling** | **Median** | | **IQR** | |
| --- | --- | --- | --- | --- | --- | --- |
|  |  |  | **Opt_EEM_** | **Opt_SC_** | **Opt_EEM_** | **Opt_SC_** |
| AD | Median | subset95 | 0 | 0 | 1.03 | 1.07 |
|  |  | subset90 | 0 | 0 | 0.90 | 1.20 |
|  |  | subset80 | 0 | 0 | 1.04 | 1.24 |
|  |  | subset70 | 0.084 | 0.17 | 0.95 | 1.28 |
|  |  | subset60 | 0.17 | 0.43 | 1.09 | 1.44 |
|  |  | subset50 | 0.36 | 0.68 | 1.08 | 1.25 |
|  | Peak Height | subset95 | -1.54 | -0.50 | 6.67 | 3.68 |
|  |  | subset90 | -4.27 | -2.23 | 3.55 | 5.75 |
|  |  | subset80 | -2.49 | -1.53 | 6.69 | 5.24 |
|  |  | subset70 | -2.17 | -1.99 | 6.80 | 4.42 |
|  |  | subset60 | -2.43 | -3.79 | 6.87 | 5.06 |
|  |  | subset50 | -4.00 | -3.48 | 7.98 | 4.35 |
|  | Peak Width | subset95 | 0 | 0 | 1.74 | 2.04 |
|  |  | subset90 | 0.58 | 0.14 | 1.41 | 2.34 |
|  |  | subset80 | 0.44 | 0.29 | 2.05 | 1.84 |
|  |  | subset70 | 0.43 | 0.14 | 2.41 | 3.37 |
|  |  | subset60 | 1.01 | 1.14 | 2.68 | 2.93 |
|  |  | subset50 | 1.03 | 1.15 | 2.30 | 3.15 |
|  | Peak Value | subset95 | -0.65 | 0.46 | 3.13 | 6.41 |
|  |  | subset90 | 1.52 | -0.36 | 6.99 | 4.67 |
|  |  | subset80 | 1.55 | 0.22 | 4.72 | 3.33 |
|  |  | subset70 | 2.00 | -0.36 | 5.03 | 5.40 |
|  |  | subset60 | 0.24 | -0.56 | 7.86 | 5.15 |
|  |  | subset50 | -0.40 | -0.004 | 5.81 | 7.29 |

**Table S6** – **The median and IQR ranges of the relative error (in percentage) of the RD histogram metrics (median, peak height, peak width, and peak value) for all subsampling methods (Opt_EEM_, Opt_SC_).**

| **Map** | **Metric** | **Subsampling** | **Median** | | **IQR** | |
| --- | --- | --- | --- | --- | --- | --- |
|  |  |  | **Opt_EEM_** | **Opt_SC_** | **Opt_EEM_** | **Opt_SC_** |
| RD | Median | subset95 | 0.37 | 0.17 | 2.12 | 2.09 |
|  |  | subset90 | 0.37 | 0.40 | 2.09 | 2.10 |
|  |  | subset80 | 0.37 | 0.40 | 1.83 | 1.84 |
|  |  | subset70 | 0.40 | 0.80 | 2.13 | 2.06 |
|  |  | subset60 | 0.60 | 1.17 | 2.10 | 2.23 |
|  |  | subset50 | 0.81 | 1.95 | 2.64 | 2.46 |
|  | Peak Height | subset95 | -0.57 | -0.00052 | 2.87 | 3.27 |
|  |  | subset90 | -0.57 | 0.12 | 2.93 | 4.00 |
|  |  | subset80 | 0.71 | -0.25 | 3.86 | 2.54 |
|  |  | subset70 | -0.26 | -0.28 | 3.91 | 3.87 |
|  |  | subset60 | -1.17 | -0.27 | 3.52 | 3.27 |
|  |  | subset50 | -0.26 | 0.89 | 3.48 | 4.28 |
|  | Peak Width | subset95 | 0.65 | 0.66 | 3.45 | 3.45 |
|  |  | subset90 | 0.65 | 0.65 | 3.28 | 3.45 |
|  |  | subset80 | 0.66 | 0.66 | 3.45 | 3.62 |
|  |  | subset70 | 0.65 | 0.67 | 2.52 | 3.56 |
|  |  | subset60 | 0 | 0 | 2.69 | 2.62 |
|  |  | subset50 | 1.00 | -0.97 | 3.05 | 3.30 |
|  | Peak Value | subset95 | -1.90 | 0.36 | 2.92 | 2.09 |
|  |  | subset90 | -1.43 | -0.40 | 3.83 | 4.83 |
|  |  | subset80 | -0.76 | -1.07 | 4.12 | 4.19 |
|  |  | subset70 | 0.20 | -0.93 | 2.09 | 3.32 |
|  |  | subset60 | -0.76 | -0.97 | 3.26 | 3.11 |
|  |  | subset50 | 0.20 | 1.46 | 2.81 | 2.77 |

**Table S7** – **The median and IQR ranges of the relative error (in percentage) of the MK histogram metrics (median, peak height, peak width, and peak value) for all subsampling methods (Opt_EEM_, Opt_SC_).**

| **Map** | **Metric** | **Subsampling** | **Median** | | **IQR** | |
| --- | --- | --- | --- | --- | --- | --- |
|  |  |  | **Opt_EEM_** | **Opt_SC_** | **Opt_EEM_** | **Opt_SC_** |
| MK | Median | subset95 | -0.28 | -0.14 | 1.38 | 1.22 |
|  |  | subset90 | -0.30 | -0.30 | 1.16 | 1.23 |
|  |  | subset80 | -0.61 | -0.61 | 1.17 | 1.08 |
|  |  | subset70 | -1.00 | -0.87 | 1.00 | 1.57 |
|  |  | subset60 | -1.45 | -1.36 | 1.36 | 1.63 |
|  |  | subset50 | -2.03 | -1.68 | 1.35 | 1.15 |
|  | Peak Height | subset95 | -2.78 | -2.22 | 5.60 | 5.77 |
|  |  | subset90 | -2.32 | -3.25 | 5.23 | 5.44 |
|  |  | subset80 | -2.96 | -3.04 | 9.56 | 5.31 |
|  |  | subset70 | -4.53 | -4.50 | 7.29 | 7.62 |
|  |  | subset60 | -4.26 | -5.27 | 7.86 | 10.21 |
|  |  | subset50 | -6.58 | -7.62 | 9.38 | 11.60 |
|  | Peak Width | subset95 | 1.49 | 0.62 | 7.41 | 7.19 |
|  |  | subset90 | 1.75 | 1.65 | 6.80 | 7.62 |
|  |  | subset80 | 2.37 | 2.23 | 7.96 | 7.09 |
|  |  | subset70 | 3.80 | 3.05 | 7.96 | 7.82 |
|  |  | subset60 | 4.60 | 4.12 | 9.42 | 8.20 |
|  |  | subset50 | 6.13 | 5.12 | 8.78 | 9.41 |
|  | Peak Value | subset95 | -0.30 | 0.42 | 2.19 | 2.81 |
|  |  | subset90 | -1.60 | -0.74 | 1.49 | 2.81 |
|  |  | subset80 | -1.25 | -1.71 | 2.06 | 2.33 |
|  |  | subset70 | 1.70 | -0.58 | 1.58 | 0.73 |
|  |  | subset60 | -2.04 | -2.02 | 2.88 | 2.04 |
|  |  | subset50 | -2.24 | -2.65 | 1.85 | 1.33 |

**Table S8** – **The median and IQR ranges of the relative error (in percentage) of the AK histogram metrics (median, peak height, peak width, and peak value) for all subsampling methods (Opt_EEM_, Opt_SC_).**

| **Map** | **Metric** | **Subsampling** | **Median** | | **IQR** | |
| --- | --- | --- | --- | --- | --- | --- |
|  |  |  | **Opt_EEM_** | **Opt_SC_** | **Opt_EEM_** | **Opt_SC_** |
| AK | Median | subset95 | 0 | 0.42 | 1.74 | 1.42 |
|  |  | subset90 | 0.42 | 0.43 | 1.69 | 1.33 |
|  |  | subset80 | 0.44 | 0.86 | 1.74 | 1.44 |
|  |  | subset70 | 1.26 | 1.31 | 1.91 | 1.45 |
|  |  | subset60 | 1.29 | 2.15 | 1.86 | 1.48 |
|  |  | subset50 | 2.09 | 3.49 | 1.80 | 1.37 |
|  | Peak Height | subset95 | -0.30 | 0.081 | 5.05 | 4.97 |
|  |  | subset90 | -2.90 | -1.69 | 5.76 | 5.28 |
|  |  | subset80 | -2.13 | -1.85 | 7.19 | 6.51 |
|  |  | subset70 | -3.10 | -3.10 | 5.64 | 5.48 |
|  |  | subset60 | -3.32 | -4.36 | 2.50 | 6.81 |
|  |  | subset50 | -3.63 | -7.26 | 6.07 | 6.28 |
|  | Peak Width | subset95 | 0.71 | 0 | 5.62 | 4.83 |
|  |  | subset90 | 1.05 | 0.72 | 5.09 | 4.59 |
|  |  | subset80 | 2.13 | 1.38 | 4.82 | 4.71 |
|  |  | subset70 | 2.73 | 2.78 | 4.40 | 4.40 |
|  |  | subset60 | 4.97 | 4.61 | 3.81 | 4.40 |
|  |  | subset50 | 6.50 | 6.43 | 4.68 | 4.13 |
|  | Peak Value | subset95 | -0.67 | -1.90 | 3.81 | 4.61 |
|  |  | subset90 | -0.42 | -1.08 | 4.90 | 3.36 |
|  |  | subset80 | -0.21 | -1.51 | 3.67 | 4.60 |
|  |  | subset70 | 0.89 | -0.41 | 4.50 | 4.81 |
|  |  | subset60 | -0.64 | 0.82 | 3.21 | 3.59 |
|  |  | subset50 | -0.65 | 0.87 | 5.71 | 4.62 |

**Table S9** – **The median and IQR ranges of the relative error (in percentage) of the RK histogram metrics (median, peak height, peak width, and peak value) for all subsampling methods (Opt_EEM,_ Opt_SC_).**

| **Map** | **Metric** | **Subsampling** | **Median** | | **IQR** | |
| --- | --- | --- | --- | --- | --- | --- |
|  |  |  | **Opt_EEM_** | **Opt_SC_** | **Opt_EEM_** | **Opt_SC_** |
| RK | Median | subset95 | -0.71 | -0.51 | 1.56 | 1.50 |
|  |  | subset90 | -0.93 | -0.81 | 1.55 | 1.38 |
|  |  | subset80 | -1.70 | -1.76 | 1.79 | 1.53 |
|  |  | subset70 | -2.77 | -3.00 | 1.45 | 1.87 |
|  |  | subset60 | -4.01 | -4.24 | 1.75 | 2.10 |
|  |  | subset50 | -5.69 | -6.28 | 1.90 | 2.19 |
|  | Peak Height | subset95 | -2.12 | -1.65 | 4.55 | 4.44 |
|  |  | subset90 | -2.83 | -3.66 | 4.78 | 3.93 |
|  |  | subset80 | -4.24 | -3.75 | 4.46 | 4.28 |
|  |  | subset70 | -5.11 | -5.24 | 7.85 | 5.72 |
|  |  | subset60 | -7.65 | -7.37 | 3.10 | 5.52 |
|  |  | subset50 | -8.32 | -11.48 | 6.97 | 6.15 |
|  | Peak Width | subset95 | 1.14 | 1.15 | 4.80 | 4.30 |
|  |  | subset90 | 1.19 | 1.44 | 4.44 | 4.80 |
|  |  | subset80 | 2.59 | 2.37 | 5.04 | 4.14 |
|  |  | subset70 | 3.88 | 4.30 | 5.23 | 4.11 |
|  |  | subset60 | 4.40 | 5.28 | 5.53 | 4.38 |
|  |  | subset50 | 5.55 | 6.82 | 5.44 | 5.69 |
|  | Peak Value | subset95 | -0.29 | 0.64 | 5.41 | 3.83 |
|  |  | subset90 | 0.31 | 0.11 | 2.99 | 5.07 |
|  |  | subset80 | -1.04 | -0.34 | 3.48 | 3.20 |
|  |  | subset70 | -2.51 | -4.50 | 7.26 | 6.99 |
|  |  | subset60 | -2.97 | -2.76 | 5.18 | 7.44 |
|  |  | subset50 | -4.99 | -4.21 | 6.00 | 6.02 |
